# The double-edged sword effect of macrophage targeting delivery system in different macrophage subsets related diseases

**DOI:** 10.1186/s12951-020-00721-3

**Published:** 2020-11-16

**Authors:** Yuchuan Yuan, Ling Long, Jiaxing Liu, Yongyao Lin, Cuiping Peng, Yue Tang, Xuemei Zhou, Shuhui Li, Chengyuan Zhang, Xiaohui Li, Xing Zhou

**Affiliations:** 1 Department of Pharmaceutics, College of Pharmacy, Army Medical University, Chongqing, 400038 China; 2 School of Pharmacy and Bioengineering, Chongqing University of Technology, Chongqing, 400054 China; 3 Department of Clinical Biochemistry, College of Pharmacy, Army Medical University, Chongqing, 400038 China; 4 Department of Oncology, Xinqiao Hospital, Army Medical University, Chongqing, 400042 China

**Keywords:** Macrophage, Targeting mechanism, Dextran modification, Macrophage related disease, Targeted delivery system

## Abstract

**Background:**

Monocyte/macrophage-targeting delivery systems (MTDSs) have been focused upon as an emerging routine for delivering drugs to treat various macrophage-related diseases. However, the ability of MTDSs to distinguish different macrophage-related diseases and their impact on macrophage function and disease progression have not been systematically revealed, which is important for actively targeted therapeutic or diagnostic strategies.

**Results:**

Herein, we used dextran-modified polystyrene nanoparticles (DEX-PS) to demonstrate that modification of nanoparticles by dextran can specifically enhance their recognition by M2 macrophages in vitro, but it is obstructed by monocytes in peripheral blood according to in vivo assays. DEX-PS not only targeted and became distributed in tumors, an M2 macrophage-related disease, but was also highly distributed in an M1 macrophage-related disease, namely acute peritonitis. Thus, DEX-PS acts as a double-edged sword in these two different diseases by reeducating macrophages to a pro-inflammatory phenotype.

**Conclusions:**

Our results suggest that MTDSs, even those designed based on differential expression of receptors on specific macrophage subtypes, lack the ability to distinguish different macrophage subtype-related diseases in vivo. In addition to the potential impact of these carrier materials on macrophage function, studies of MTDSs should pay greater attention to the distribution of nanoparticles in non-target macrophage-infiltrated disease sites and their impact on disease processes.
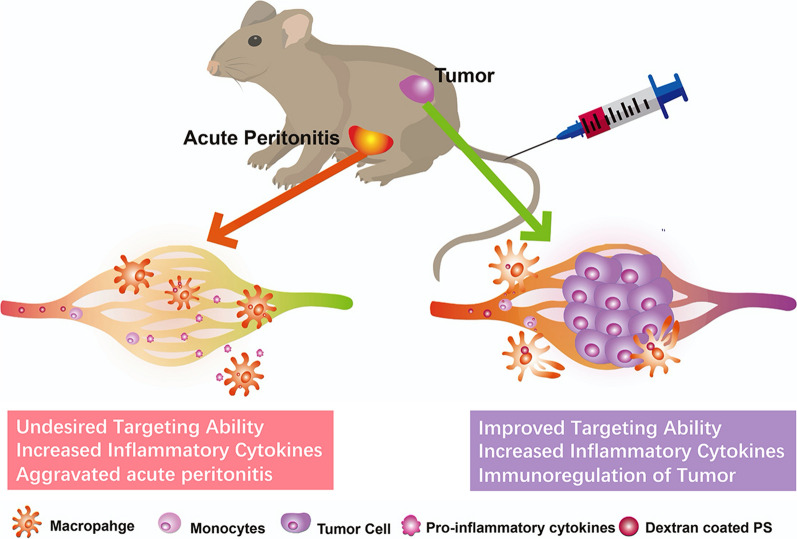

## Background

Inspired by the recruitment of monocytes/macrophages in various diseases, a tremendous number of macrophage/monocyte-targeted delivery systems (MTDSs) have been engineered using targeting motifs and applied in various macrophage-related diseases, such as atherosclerosis [1–4], inflammation [5–7], and especially cancer [8–10]. This widespread availability of MTDSs in various diseases is not only an advantage but also a potential risk. For example, tumor patients often present with a variety of inflammation-related complications [11–14]. In such cases, the MTDS may not only target and deliver drugs to the local tumor but also deliver drugs to other inflammation-related disease sites or lesions. However, treatments applied for tumors are far removed from those used to treat inflammation. Whether a chemotherapeutic that plays a cytotoxic role or an immunotherapy agent that promotes local inflammatory responses to the tumor, undesired delivery of these drugs to inflammatory lesions may bring about serious injury for a range of inflammation-related diseases.

In addition to the influence of loaded drugs on diseases, researchers have found that biomaterials and nanovehicles can also participate in therapeutic mechanisms such as regulation of reactive oxygen species (ROS) or immunity [15–17]. Although a terminally differentiated cell type, the functional state of macrophages is plastic in response to different stimuli [18, 19]. In general, macrophages are classed as two distinct phenotypes, pro-inflammatory macrophages (M1 macrophages) and anti-inflammatory macrophages (M2 macrophages). Despite many of these functions benefit the protecting of host, the uncontrolled number and excessive activation of macrophages play great roles in the development of many diseases, such as diabetes [20], inflammation [21], atherosclerosis [22], and cancer [23]. In tumor, macrophages infiltrate into the tumor stroma as tumor-associated macrophages (TAMs), and then most of them activate to M2-like macrophages for construct a supportive microenvironment to promote tumor growth. On the other hand, uncontrolled recruitment of M1-like macrophages in local of inflammation can aggravate numerous inflammation diseases, such as peritonitis, rheumatoid arthritis, obesity, diabetes, atherosclerosis, and myocardial infarction [24]. Some biomaterials and nanoparticles (NPs) have been shown to regulate macrophage function to anti-inflammation or pro-inflammation direction, by activating specific receptors on their surface [16, 25], which further magnifies the risks derived from a lack of specific targeting by an MTDS to an undesired disease.

Based on the different subtype macrophages distributed in inflammation lesions and tumor, many researchers intended to precisely target either a tumor or inflammation-related disease by employing targeting motifs to recognize a specific antigen or receptor on M1 [26, 27, 28] or M2 macrophages [29, 30, 31], however, the ability of these systems to distinguish between M1 and M2 macrophage subtype-related diseases was not evaluated in these studies. Moreover, such strategies do not seem reliable. Dextran is a specific ligand of macrophage mannose receptor (MMR) and scavenger receptor [32]. Both MMR and scavenger receptor are especially highly expressed on M2 macrophages, and lowly expressed on M1 macrophages, however, a puzzling phenomenon is that dextran were successfully employed as a targeting unit not only for targeting tumors, but also for inflammatory diseases [33, 34, 35, 36, 37]. Importantly, whether the specific recognition of MMR on the surface of M2 macrophages by dextran cannot distinguish inflammation from tumor, as well as its mechanism and potential risks still unrevealed.

As a M2 macrophage related disease, breast cancer is a common tumor model for studying macrophage targeted drug delivery system, in which TAMs are the major tumor microenvironment (TME) component [38]. Meanwhile, as a M1 macrophage related disease, peritonitis is a typical disease model employed in studying the relationship between macrophages and inflammation, which holds a large number of peritoneal macrophages [39] and is also a kind of complication caused by breast cancer and other tumor metastasis to abdominal cavity [39, 40]. Herein, to elaborate the distribution and disease influence of dextran-modified NPs, we systematically evaluated the targeting ability and effects of dextran-functionalized polystyrene NPs (DEX-PS) to different macrophage subtypes in vitro and in vivo, employing breast cancer and peritonitis as representative M2 and M1 macrophage related diseases.

## Result and discussion

### In vitro targeting ability of DEX-PS to M2 macrophages

Nile Red-labeled polystyrene NPs 500 nm in size and with similar fluorescence properties, size distribution, and surface potential (Additional file 1: Figure S1a–c, S1e–g), including DEX-PS, carboxyl-functionalized polystyrene NPs (COOH-PS), and unfunctionalized polystyrene NPs (PS), were employed in this study to ensure that NPs could reach the disease focus through the enhanced permeability and retention effect [41, 42] and only be specifically phagocytosed by macrophages [43, 44, 45]. DEX -PS were synthesised by COOH-PS and Amine-dextran under condensation reaction. Compared with COOH-PS, both dextran-NH_2_ and DEX-PS showed an obvious hydroxyl peak in dextran at 3307 cm^−1^, indicating that dextran has been successfully modified on COOH-PS to form DEX-PS (Additional file 1: Figure S1d). By measuring the residual content of dextran-NH_2_ in the supernatant after reaction, we also determined that the dextran content modified on DEX-PS was 17.1% ± 0.03%.

NPs were proven to not be cytotoxic to RAW264.7 cells (a murine monocyte/macrophage cell line) at particle-to-cell ratios in the range of 6.25–100 (Additional file 1: Figure S2a), while showing similar fluorescent intensities at an identical concentration (Additional file 1: Figure S2b). Thus, PS, COOH-PS, and DEX-PS at a particle-to-cell ratio of 100 were employed in further experiments. M1 and M2 macrophages were polarized from RAW264.7 cells by stimulation with LPS and IFN-γ or IL-4, respectively, and verified by the determination of surface markers, cytokines, and morphological characteristics by flow cytometry, enzyme-linked immunosorbent assay, PCR, and phase-contrast microscopy (Additional file 1: Figure S3). Because of the strong phagocytosis ability of macrophages, the rate of uptake of NPs by macrophages approached 100% after co-incubation for 24 h (Additional file 1: Figure S2c). Hence, the incubation time for uptake in this study was limited to less than 4 h to observe differences in the phagocytic ability of macrophages towards different polystyrene NPs.

**Fig. 1:**
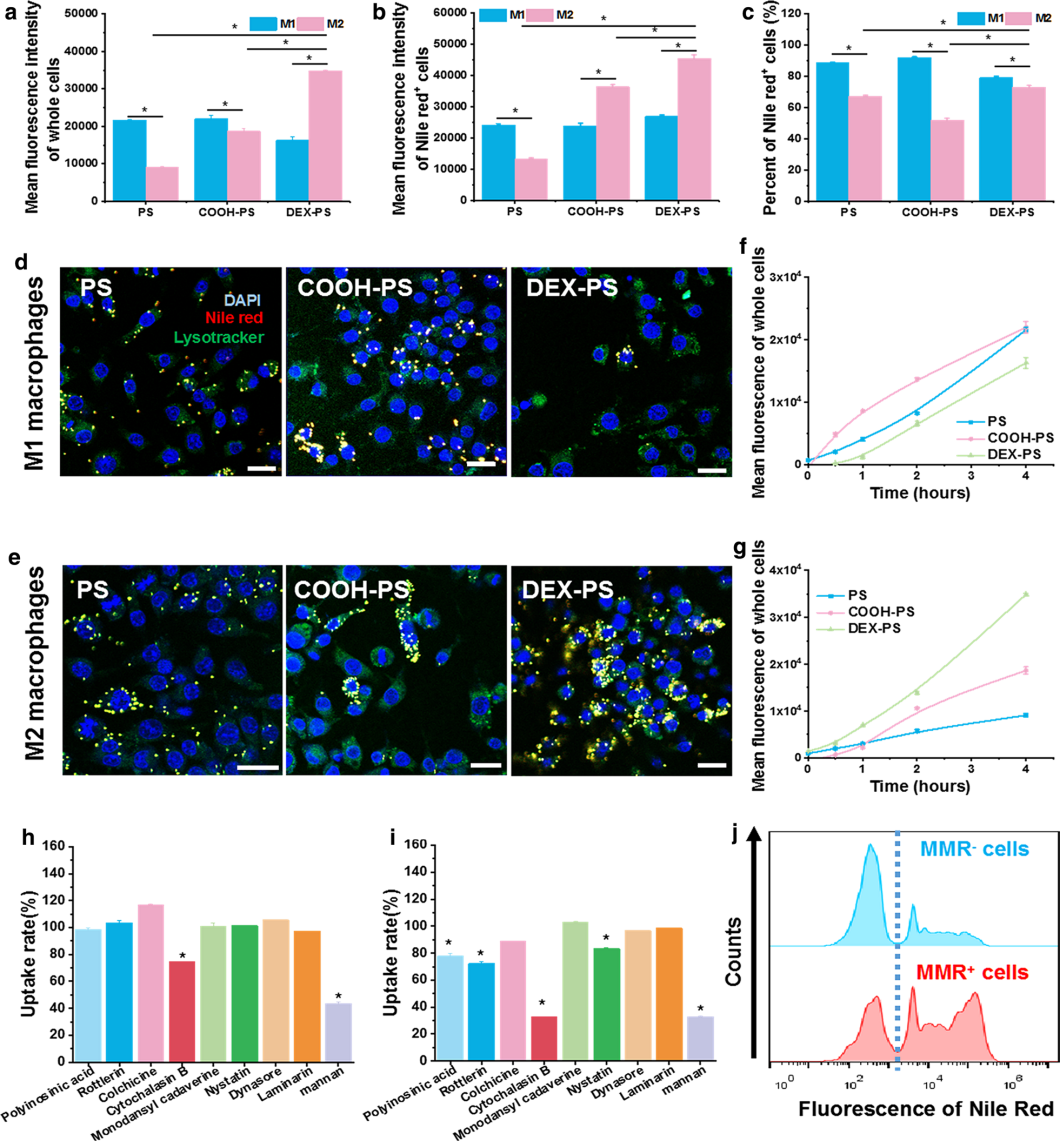
DEX-PS uptake behavior by different macrophage subtypes. Mean fluorescence of whole cells (**a**) and Nile Red^+^ cells (**b**), and percentages of Nile Red^+^ cells (**c**) after incubation with various NPs. Representative confocal laser-scanning microscopy images of M1 (**d**) and M2 macrophages (**e**) incubated with PS, COOH-PS, or DEX-PS for 4 h. Kinetic profile of mean fluorescence of whole M1 (**f**) and M2 macrophages (**g**). Rate of DEX-PS uptake by M1 (**h**) and M2 macrophages (**i**) influenced by various inhibitors. **j** Fluorescence distribution of DEX-PS in MMR^+^ and MMR^−^ cells. Scale bars in **d** and **e** indicate 20 µm. Data are presented as the mean ± SD (n = 3), * Indicates p < 0.05. COOH-PS: carboxyl-functionalized polystyrene; DEX-PS: dextran-functionalized polystyrene; MMR: macrophage mannose receptor; PS: polystyrene

To verify the ability of the dextran modification to improve targeting of NPs to M2 macrophages, we first monitored the internalization process of various NPs by M1 and M2 macrophages. After incubation with various NPs for 4 h, total uptake of DEX-PS by M2 macrophages, as reflected by the mean fluorescence of whole cells, was much higher compared with other NPs (Fig. 1a). Although uptake of PS and COOH-PS by M1 macrophages was significantly higher than that of M2 macrophages, the dextran modification reversed this trend as phagocytosis, as indicated by a significant increase of DEX-PS in M2 macrophages compared with M1 macrophages (Fig. 1a). Moreover, our analysis of proportions of macrophages that ingested NPs and the average fluorescence intensity of cells that phagocytized NPs suggested that the M2-specific phagocytic characteristics brought about by the dextran modification mainly arose from enhanced phagocytic ability of individual M2 macrophages for NPs (Fig. 1b). In contrast, the observed decrease in phagocytosis of M1 macrophages caused by dextran modification mainly resulted from a decreased proportion of cells involved in phagocytosis of DEX-PS (Fig. 1c). These findings were further verified by fluorescent images obtained at 4 h (Fig. 1d, e) and were reproduced in bone marrow derived macrophage cells (Additional file 1: Figure S4). In addition, uptake of DEX-PS by M1 macrophages was lower than that of PS and COOH-PS at all time points, while that of M2 macrophages was increased compared with PS and COOH-PS at all-time points (Fig. 1f, g).

To reveal the mechanism underlying improved internalization of DEX-PS by M2 macrophages, the internalization mechanisms of DEX-PS, COOH-PS, and PS by M1 and M2 macrophage subtypes were examined. After pretreating macrophages with various uptake inhibitors prior to incubation with polystyrene NPs, we found that M1 macrophages took up DEX-PS mainly via cytochalasin B-inhibited phagocytosis and an MMR-dependent uptake pathway (Fig. 1h), whereas M2 macrophages took up DEX-PS via a scavenger receptor-dependent pathway, phagocytosis, macropinocytosis, caveolae-mediated endocytosis, and primarily an MMR-dependent uptake pathway (Fig. 1i). Under stimulation by dextran, MMR expression on M1 macrophages was greatly improved after incubation with DEX-PS for 4 h (Additional file 1: Figure S5), which explained the increased rate of DEX-PS uptake by M1 macrophages after incubation for 1 h. In contrast, uptake of unmodified PS and COOH-PS by M1 and M2 macrophages was more irregular and occurred by a combination of multiple mechanisms (Additional file 1: Figure S6).

To further verify the role of MMR on uptake behavior of DEX-PS by macrophages, MMR expression was analyzed after incubating M2 macrophages with DEX-PS for 4 h (Fig. 1j, Additional file 1: Figure S7). The proportion and average fluorescence intensity of Nile Red-positive (Nile Red^+^) cells in MMR^+^ macrophages were much higher than observed in MMR^−^ macrophages (Fig. 1j, Additional file 1: Figure S7). These results suggest that MMR^+^ macrophages were more likely and had a higher capacity to take up DEX-PS than MMR^−^ macrophages, further verifying that improved uptake of DEX-PS by M2 macrophages mainly arose from specific recognition of the mannose receptor [46]. Because MMR expression in M2 macrophages was much higher than observed in M1 macrophages (Additional file 1: Figure S3), DEX-PS was specifically phagocytosed by M2 macrophages in vitro.

### In vivo targeting ability of DEX-PS to M1 and M2 macrophage subtype-related diseases

It is of great importance to verify that the specificity of NP internalization by M2 macrophages in vitro can be reproduced in vivo, so that NPs can precisely distribute in M2-related tumors rather than other inflammatory areas primarily infiltrated by M1 macrophages.

Then, the in vivo distribution of NPs in acute peritonitis and tumors was we further observed. To avoid interference derived from different clearance rates of various NPs in peripheral blood, the time point of 8 h after intravenous (i.v.) injection of NPs was employed to observe their in vivo distribution. At that time, no significant difference of Nile Red fluorescence intensity was found between groups (Additional file 1: Figure S8).

**Fig. 2:**
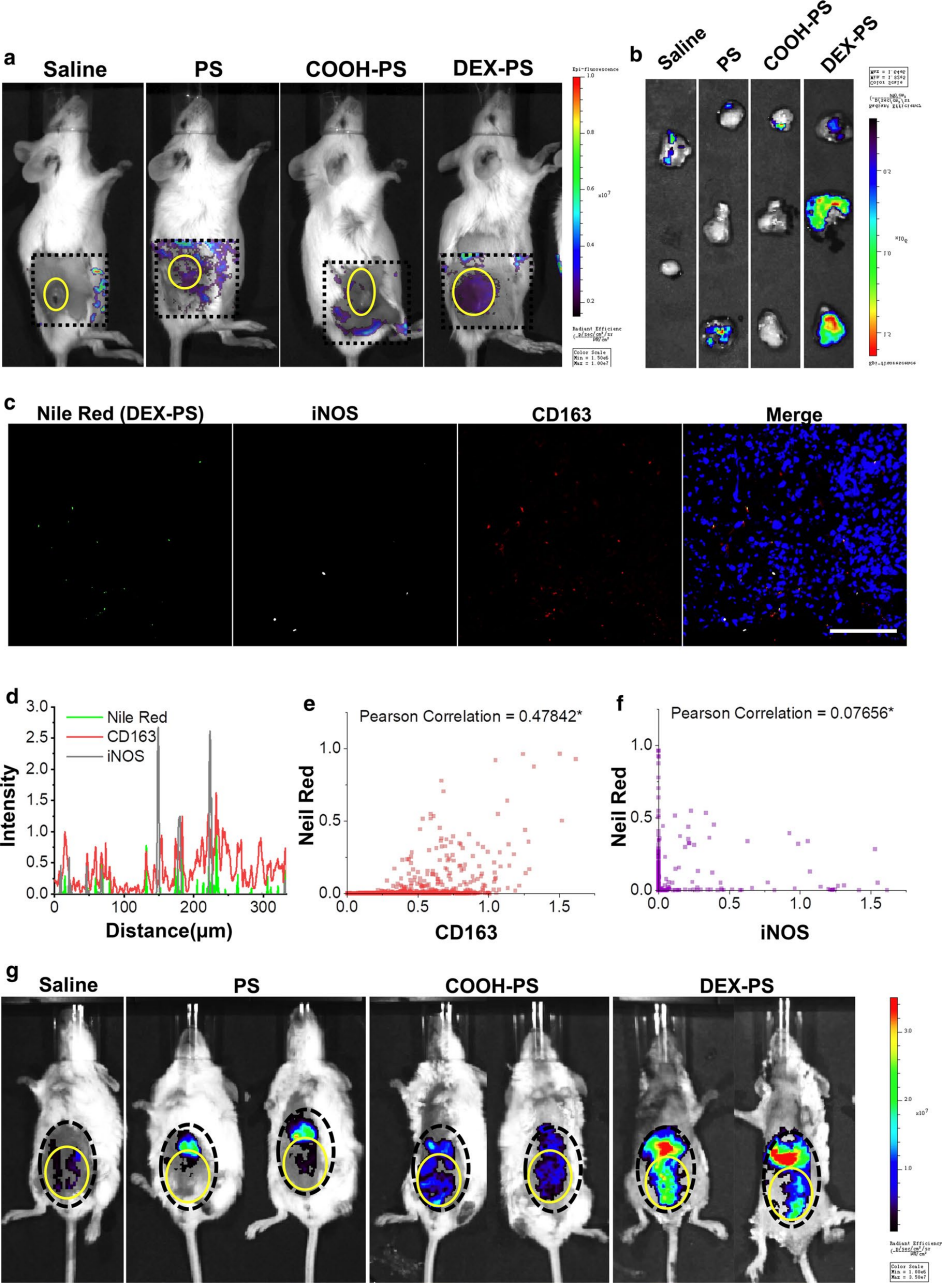
In vivo targeting ability of DEX-PS to tumor and acute peritonitis. **a** Typical in vivo images illustrating Nile Red fluorescence signals in tumor-bearing mice. **b** Ex vivo images illustrating Nile Red fluorescence signals in isolated tumors. **c** Distribution of nanoparticles in tumor section observed by confocal laser-scanning microscopy. **d** Colocalization analysis of nanoparticles with iNOS and CD163. **e** Pearson correlation assay of nanoparticles and CD163. **f** Pearson correlation assay of nanoparticles and iNOS. **g** Typical in vivo distribution of Nile Red fluorescent signals in acute peritonitis. The area marked by the dotted black line is the focus of disease, while the area marked by the solid yellow line is the region of interest. COOH-PS, carboxyl-functionalized polystyrene; DEX-PS: dextran-functionalized polystyrene; iNOS: inducible nitric oxide synthase; PS: polystyrene

Firstly, the mainly macrophages subtypes distributed in mice 4T1 breast tumor was verified to be M2 macrophage (Additional file 1: Figure S9a). As expected, obviously enhanced fluorescence signals were observed in tumors of mice treated with DEX-PS both in vivo and ex vivo (Fig. 2a, b), while both COOH-PS and PS failed to target tumor lesions. In addition, DEX-PS exhibited high distributions in the liver, spleen, and lung, while PS and COOH-PS showed much weaker fluorescence in these organs (Additional file 1: Figure S10c). Further observation revealed that DEX-PS in tumors was mainly distributed in M2 macrophages (Fig. 2c–f), indicating that DEX-PS successfully reached the tumor site by targeting M2 macrophages. However, we found that the active-targeting ability of DEX-PS to M2 macrophages in vitro did not avoid distribution of DEX-PS in acute peritonitis, in which we have verified that the macrophages were M1-like status (Additional file 1: Figure S9b). Indeed, DEX-PS showed good targeting ability to acute peritonitis (Fig. 2g, Additional file 1: Figure S10b). In contrast, PS and COOH-PS showed no significant fluorescence at peritonitis lesions, similar to tumor-bearing mice (Fig. 2a). These results suggest that DEX-PS, which could be specifically recognized by M2 macrophages in vitro, lacked the ability to precisely distinguish between different macrophage-subtype related diseases in vivo.

### In vivo fate of DEX-PS in peripheral blood

**Fig. 3:**
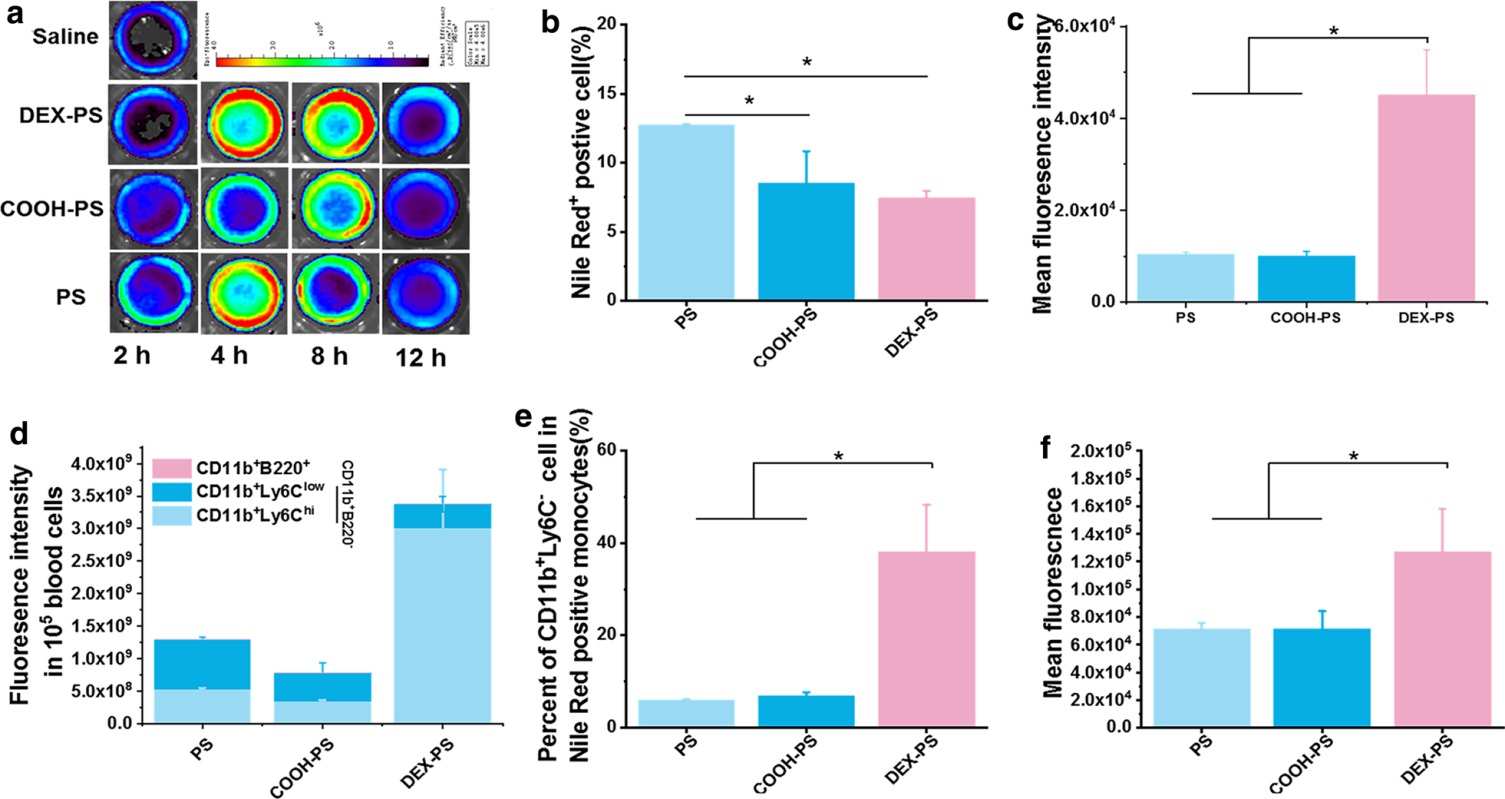
In vivo fate of DEX-PS in peripheral blood. **a** Ex vivo images illustrating Nile Red fluorescence signals in isolated whole blood. **b** Percentages of Nile Red-positive blood cells. **c** Mean and **d** total fluorescence intensity of Nile Red in Nile Red^+^ cells. **e** Percentages of CD11b^+^/Ly6C^hi^ cells in Nile Red^+^ monocytes (CD11b^+^/B220^−^). **f** Mean fluorescence intensity of CD11b^+^/Ly6C^hi^ subset of Nile Red^+^ monocytes. Data are presented as the mean ± SD (n = 3), * Indicates p < 0.05. COOH-PS: carboxyl-functionalized polystyrene; DEX-PS: dextran-functionalized polystyrene; PS: polystyrene

To understand the inconsistent behavior of DEX-PS in vivo and in vitro, we further investigated the fate of NPs after i.v. injection by monitoring changes in fluorescence intensity of peripheral blood derived from mice treated with various NPs over the first 12 h (Fig. 3a). Despite PS-treated mice exhibiting much higher peak fluorescence intensities, COOH-PS- and DEX-PS-treated mice exhibited more stable peak fluorescence intensities, with only a tiny descent in the first 8 h compared with the sharp descent observed at 8 h in the PS-treated group (Additional file 1: Figure S8). These results indicate that DEX-PS was cleaned up more slowly in peripheral blood compared with other NPs.

Blood cells of DEX-PS-treated mice showed the lowest number of Nile Red^+^ cells among the three groups (Fig. 3b); however, mean fluorescence intensity in this group was much higher than observed in PS- or COOH-PS treated mice (Fig. 3c). Accordingly, the total fluorescence intensity of positive cells in the DEX-PS group was much higher than observed in the other groups (Fig. 3d), and these positive cells were mainly blood monocytes (CD11b^+^/B220^−^) (Additional file 1: Figure S11). Correspondingly, Nile Red fluorescence was mainly displayed in blood monocytes, with small amounts distributed in B lymphocytes (CD11b^+^/B220^+^) (Fig. 3d). Furthermore, there was no difference between the distributions of PS and COOH-PS in Ly6C^hi^ and Ly6C^low^ monocytes, while DEX-PS was obviously phagocytized more by Ly6C^hi^ monocytes than Ly6C^low^ monocytes (Fig. 3d). Moreover, compared with PS and COOH-PS, more Ly6C^hi^ monocytes were involved in phagocytosis of DEX-PS (Fig. 3e), and the average fluorescence intensity of these monocytes was much higher (Fig. 3f).

Macrophages localized in lesions are usually derived and activated from Ly6C^hi^ monocytes in peripheral blood; accordingly, it has been shown that many kinds of NPs can be delivered to lesions under the assistance of Ly6C^hi^ monocytes [8]. Hence, the reason that DEX-PS was delivered to both tumors and acute peritonitis in vivo was probably due its high rate of uptake by Ly6C^hi^ monocytes in peripheral blood, rather than recognition by M2 macrophages in lesions.

### In vitro study on enhanced internalization of DEX-PS by monocytes

**Fig. 4:**
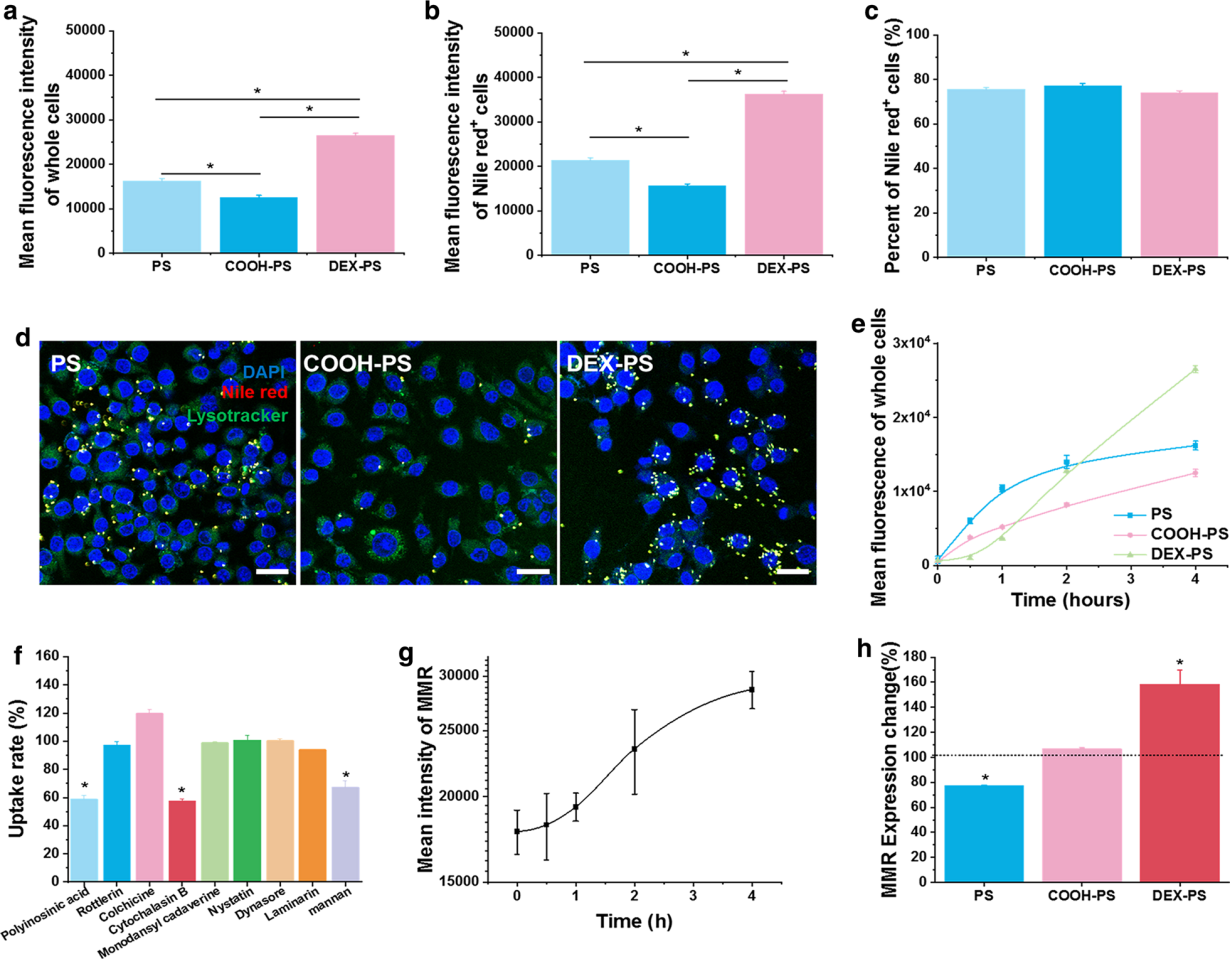
Improved uptake of DEX-PS by RAW264.7 cells. Mean fluorescence of whole cells (**a**) and Nile Red^+^ cells (**b**), and the percentage of Nile Red^+^ cells (**c**) after incubation with various NPs. **d** Confocal laser-scanning microscopy images of RAW264.7 cells after incubation with PS, COOH-PS, or DEX-PS for 4 h. **e** Kinetic profile of mean fluorescence of whole RAW264.7 cells. **f** Rates of DEX-PS uptake by RAW264.7 cells were influenced by various inhibitors. **g** Dynamic changes in mean intensity of MMR on RAW264.7 cells after incubation with DEX-PS. **h** Relative expression changes of MMR on RAW264.7 cells after 4-h incubation with PS, COOH-PS, or DEX-PS. Scale bars in **d** indicate 20 µm. Data are presented as the mean ± SD (n = 3), * Indicates p < 0.05. COOH-PS, carboxyl-functionalized polystyrene; DEX-PS: dextran-functionalized polystyrene; MMR: macrophage mannose receptor; PS: polystyrene

To further understand how dextran modification affected the ability of monocytes to uptake NPs, we evaluated engulfment of NPs by undifferentiated RAW264.7 cells (to simulate monocytes). After incubation with various NPs for 4 h, the total amount of DEX-PS uptaken by RAW264.7 cells was much higher compared with PS and COOH-PS (Fig. 4a), which was mainly attributed to the improved phagocytic ability of individual RAW264.7 cells for DEX-PS (Fig. 4b). No differences in percentages of Nile Red^+^ cells were found between DEX-PS and other groups (Fig. 4c). These findings were further verified by fluorescence images obtained at 4 h (Fig. 4d). Additionally, although total amounts of PS within cells suggested that it was initially internalized with high efficiency in the first 2 h, uptake gradually slowed afterwards. In contrast, DEX-PS within RAW264.7 cells sharply increased after incubation for 1 h, and finally peaked at a higher level than observed in cells incubated with PS (Fig. 4e). These results suggest that early engulfment of DEX-PS by RAW264.7 cells may in turn promote further uptake of DEX-PS. In contrast, uptake of PS and COOH-PS by a single RAW264.7 cell tended to saturate with time.

We next examined the mechanisms by which NPs were endocytosed by RAW264.7 cells. RAW264.7 cells mainly took up DEX-PS by a scavenger receptor and MMR-dependent pathway, and cytochalasin B-inhibited phagocytosis (Fig. 4f).

Interestingly, after incubation with DEX-PS for 4 h, MMR expression of RAW264.7 cells was significantly upregulated (160%) with time (Fig. 4g, h). In contrast, internalization mechanisms of PS and COOH-PS by RAW264.7 cells involved various pathways, excluding the MMR pathway (Additional file 1: Figure S12). Meanwhile, no obvious changes of MMR expression were observed in RAW264.7 cells incubated with PS or COOH-PS (Fig. 4h). Taken together, although MMR expression in RAW264.7 cells that did not differentiate into macrophages was very low, under stimulation with DEX-PS, MMR expression was upregulated to improve phagocytosis of DEX-PS by the MMR pathway. These results explain the strong ability of monocytes to phagocytose DEX-PS in vivo.

### In vitro effect of DEX-PS on macrophage function

**Fig. 5:**
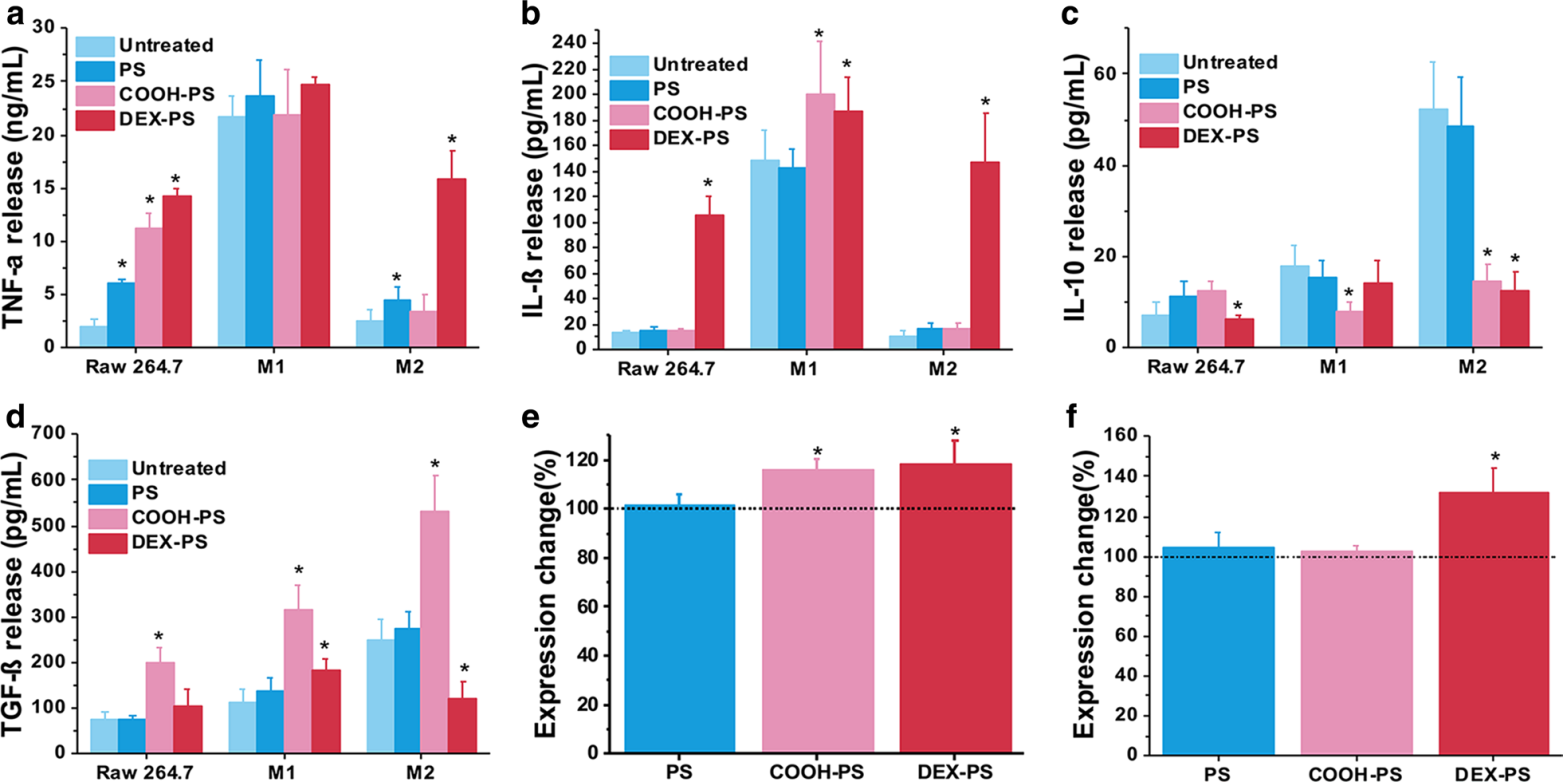
Regulation of various polystyrene nanoparticles on macrophage function. **a**–**d** Typical cytokine expression in RAW264.7 cells, and M1 and M2 macrophages. **e**, **f** Change in expression of CD86 on RAW264.7 cells (**e**) and M2 macrophages (**f**) after treatment with various nanoparticles. Data are presented as the mean ± SD (n = 3), * Indicates p < 0.05, vs. untreated. COOH-PS: carboxyl-functionalized polystyrene; DEX-PS: dextran-functionalized polystyrene; iNOS: inducible nitric oxide synthase; PS: polystyrene; TGF-β: transforming growth factor beta; TNF-α: tumor necrosis factor alpha

Both the MMR-pathogen interaction and activation of scavenger receptors [47] can initiate series of signaling pathways that direct the production of lysosomal enzymes [48], ROS [49], and pro-inflammatory cytokines such as interferon gamma (IFN-γ), tumor necrosis factor alpha (TNF-α), and interleukin 12 (IL-12) [50, 51]. Hence, the effect of DEX-PS, COOH-PS, and PS on macrophage function was monitored. DEX-PS elicited an obvious effect on monocytes/macrophages, as indicated by significant stimulation of RAW264.7 cells, and M1 and M2 macrophages to release pro-inflammatory cytokines TNF-α and interleukin 1 beta (IL-1β) (Fig. 5a, b), as well as inhibition of M2 macrophages to secrete anti-inflammatory cytokines interleukin 10 (IL-10) and transforming growth factor beta (TGF-β) (Fig. 5c, d). In contrast, although COOH-PS could stimulate increased IL-1β expression in M2 macrophages (Fig. 5b), it also increased TGF-β expression in various macrophages (Fig. 5d), which did not represent a change in their anti-inflammatory or pro-inflammatory function. In addition, after incubation with DEX-PS for 4 h, CD86 expression on RAW264.7 cells and M2 macrophages was obviously upregulated (Fig. 5e, f); in contrast, DEX-PS had no significant effect on CD86 expression of M1 macrophages (Additional file 1: Figure S13). These results suggest that macrophages tend to exert pro-inflammatory functions after ingesting DEX-PS, as polarized M2 macrophages and RAW264.7 cells were gradually transformed into M1-like macrophages with pro-inflammatory function.

### In vivo effect of DEX-PS on M1 and M2 macrophage subtype-related diseases

**Fig. 6:**
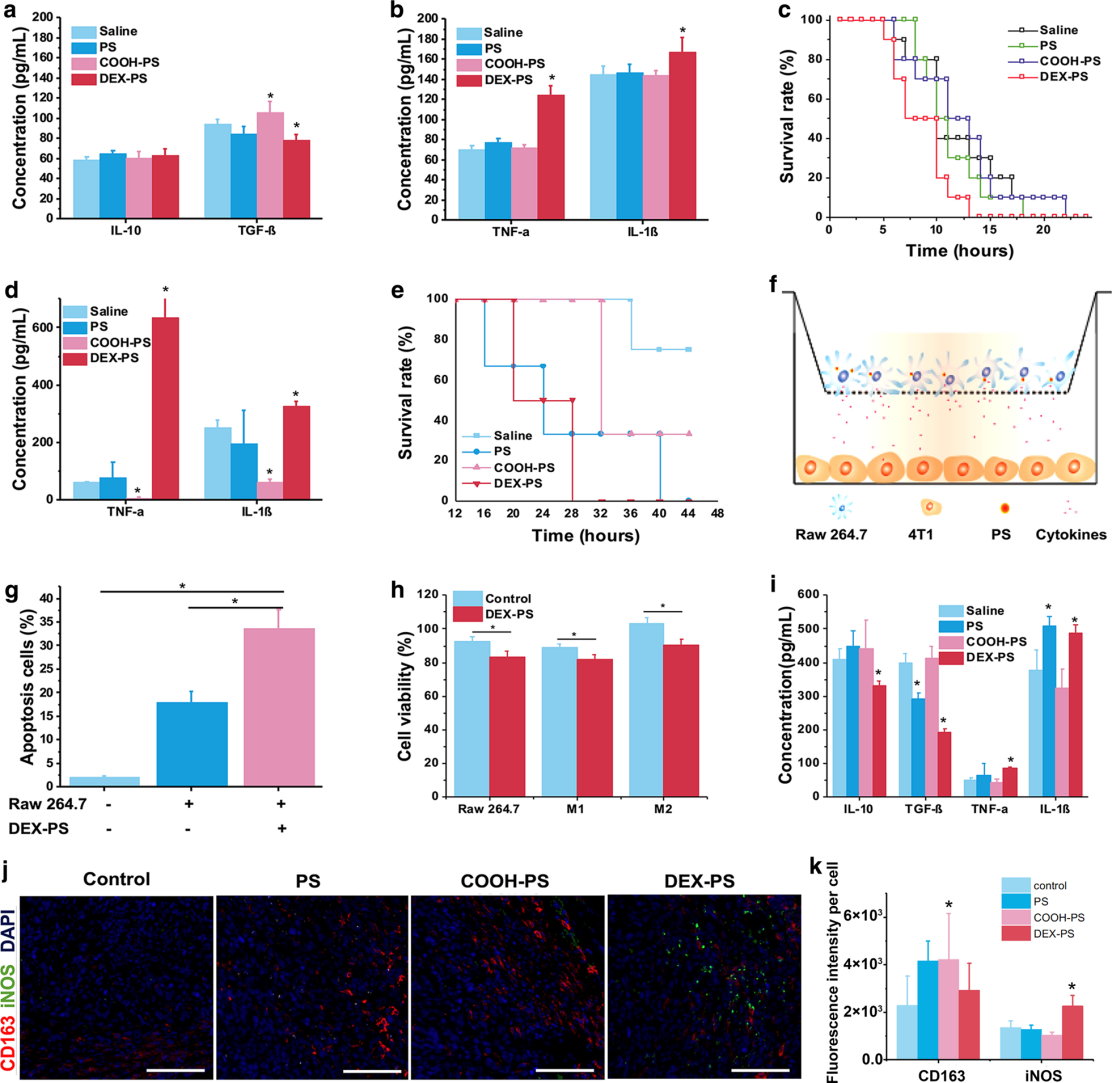
The effect of DEX-PS on progression of macrophage-related diseases. Typical cytokine expression (**a**, **b**) in abdominal dropsy and survival rate (**c**) of mice administered various NPs after peritonitis induced by i.p. injection of zymosan (n = 10). Typical cytokine expression (**d**) in abdominal dropsy and survival rate (**e**) of mice administered various NPs after peritonitis induced by i.p. injection of acetic acid (n = 5). **f** Schematic of macrophage-tumor cell co-culture system. **g** Proportion of apoptotic 4T1 tumor cells in the co-culture system determined by TUNEL assay (n = 3). **h** Viability of 4T1 tumor cells in various co-culture systems (n = 3). **i** Typical cytokine expression in tumors isolated from 4T1-bearing mice treated with various NPs (n = 6). **j** Typical immunofluorescence images of tumors isolated from 4T-bearing mice treated with various NPs. **k** Quantification analysis of fluorescence intensity of each cell (n = 3). * In **a**, **c**, **i** and **k** indicates p < 0.05, vs. saline. Data are presented as the mean ± SD, * In **g** and **h** indicates p < 0.05. Scale bar indicates 100 µm. COOH-PS: carboxyl-functionalized polystyrene; DEX-PS: dextran-functionalized polystyrene; IL-1β: interleukin 1 beta; IL-10: interleukin 10; iNOS: inducible nitric oxide synthase; PS: polystyrene; TGF-β: transforming growth factor beta; TNF-α: tumor necrosis factor alpha

Acute peritonitis is a highly lethal M1 macrophage-related disease. A zymosan-induced acute peritonitis model and acetic acid-induced acute peritonitis model were both employed to evaluate the effects of DEX-PS on these inflammatory macrophage-related diseases. In mice with zymosan-induced acute peritonitis, the degree of peritoneal inflammation was obviously enhanced by DEX-PS, as indicated by significant upregulation of TNF-α and IL-1β in the peritoneal cavity (Fig. 6a, b). As a result, the survival rate of DEX-PS-treated mice was lower than the other groups (Fig. 6c), and they had the shortest average survival time of all groups (Additional file 1: Figure S14); in contrast, neither PS or COOH-PS influenced the survival time of mice (Fig. 6c, Additional file 1: Figure S14). Similar results were observed in the acute peritonitis model induced by acetic acid, whereby DEX-PS significantly increased TNF-α and IL-1β expression in extracted abdominal dropsy (Fig. 6d), and hence speeded the death of mice (Fig. 6e). These results confirmed that DEX-PS could significantly aggravate local inflammation by regulating macrophages, which occurred because of non-specific transport by monocytes in vivo. Therefore, when dextran is employed for targeting therapy (e.g. for tumors), the potential pro-inflammatory effect of dextran-coated NPs in inflammation-related complications should be a top concern in addition to focusing on its targeting efficiency.

In contrast to acute inflammation, inflammatory macrophages play an active role in inhibiting tumor progression. Therefore, we speculated that the regulatory effect of DEX-PS on macrophages might be beneficial for cancer therapy. First, the effect of DEX-PS-treated macrophages was evaluated in vitro in a RAW264.7-4T1 co-culture system (Fig. 6f). After 24 h, RAW264.7 cells treated with DEX-PS exhibited a much higher proportion of apoptotic 4T1 cells compared with PS-treated cells (Fig. 6g). Accordingly, the viability of 4T1 cells after incubation with DEX-PS-treated RAW264.7 cells was lower than that of cells incubated with untreated RAW264.7 cells (Fig. 6h). These results suggest that DEX-PS uptake might lead macrophages to be pro-inflammatory by releasing TNF-α and IL-1β, which would induce apoptosis of tumor cells. Furthermore, after 16 days of DEX-PS treatment in Balb/C mice bearing 4T1 tumors, although only a little inhibition effect of DEX-PS on tumor growth without statistical significance was observed (Additional file 1: Figure S15), expression of pro-inflammatory cytokines TNF-α and IL-1β was increased in tumors, while IL-10 and TGF-β levels were decreased (Fig. 6i). Although changes of inflammatory cytokines were also observed in tumors of PS- or COOH-PS-treated mice, they varied between pro-inflammatory and anti-inflammatory orientations (Fig. 6i). In addition, the proportion of M1 macrophages in tumors treated with DEX-PS was much higher than observed in other groups (Fig. 6j). More importantly, although DEX-PS was initially distributed in M2 macrophages expressing a high degree of CD163 (Fig. 3c), high amounts of DEX-PS were found in M1 macrophages that highly expressed iNOS (Additional file 1: Figure S16). These results suggest that DEX-PS can reeducate tumor-associated macrophages to a pro-inflammatory phenotype, and thus play a regulatory role in the immune microenvironment of tumors.

In summary, we demonstrated that DEX-PS exerts a double-sword effect in different macrophage subtype-related diseases in that it aggravated local inflammation in acute peritonitis but reconstructed a pro-inflammatory microenvironment in tumors.

## Conclusion

In conclusion, we showed that proper surface engineering of polystyrene NPs with a specific ligand, dextran, can specifically enhance the recognition of NPs by M2 macrophages, rather than M1 macrophages, in vitro via MMR. However, the capability of NPs to distinguish between M1 and M2 macrophage subtype-related diseases in vivo was obstructed by Ly6C^hi^ monocytes in peripheral blood. Under transportation by these monocytes, DEX-PS was delivered not only to M2 macrophage-related tumors but also to M1 macrophage-related acute peritonitis. More importantly, dextran modification stimulated macrophages to release more pro-inflammatory cytokines thus reconstructing a pro-inflammatory microenvironment in tumors that is beneficial to tumor therapy, but further exacerbated local inflammation such as acute peritonitis. In addition, surface modification of NPs may lead to unexpected regulation of macrophage function, resulting in positive or negative roles in disease progression. Hence, it is of great necessity and importance to investigate the complex relationship between vehicles and monocytes/macrophages when designing an optimal drug delivery system for a specific macrophage-related disease, which is essential for actively targeted therapeutic or diagnostic strategies.

## Methods

### Materials

Nile red labeled polystyrene NPs (PS), carboxy functionalized polystyrene NPs (COOH-PS) at a mean diameter of 0.50 μm in a 1% aqueous suspension were purchased from Spherotech, Inc. (Lake Forest, IL, USA). The fluorescence excitation and emission spectra maxima were all at 517 nm and 560 nm, respectively. All cell culture media were obtained from Gibco/Life Technologies. Dextran amine at MW of 6 k Da, was purchased from Creative PEGWorks (Chapel Hill, NC, USA). Other chemical reagents used for NP synthesis were purchased from Sigma-Aldrich (St Louis, MO, US).

### Synthesis of dextran modified polystyrene NPs

COOH-PS at number of 2.5 × 10^6^ and 5 nmol of dextran amine were co-dispersed in 100 μL of 0.1 M 2-*N*-morpholino ethane sulfonic acid (MES) solution. Then 0.3 mg of EDC (1-(3-Dimethylaminopropyl)-3-ethylcarbodiimide hydrochloride) were twice added in, with a vortexing and incubating process of 20 min at ambient temperature for each time. After incubating for another 80 min on a rotary mixer, the product was centrifuge and remove the supernatant carefully. Then the obtained pellet was suspended in 1 mL of 0.1 M PBS containing 0.02% Tween-20.

### Kinetics of internalization of various NPs by macrophages

Phagocytosis assays of different phenotype macrophage were performed by incubating different surface functioned NPs with Raw 264.7 cells, M1 macrophages or M2 macrophage. The polarization from Raw 264.7 cells to M1 or M2 macrophages were verified by the determination of surface markers, cytokines and morphological characteristics by flow cytometry (FC), ELISA, PCR and phase contrast microscopy. As the fluorescence properties of various NPs, including fluorescence intensity at the same concentration, have no significant difference, the concentration of NPs employed here was fixed on the particle number/cells ratio of 100. Because the uptake rate of macrophages towards NPs approached 100% after co-incubation for 24 h, the incubation time for uptake was limited to less than 4 h. After incubating at 37  °C for 0.5, 1, 2 and 4 h, quantification of phagocytosis by FC was performed and analyzed after the macrophages were washed and detached by the FACS Aria II flow cytometer (BD) for median fluorescence intensity of the cell population in the PE channel. In addition, macrophages incubated with various NPs for 4 h were collected and observed by Zeiss LSM 710 laser-scanning microscope (Carl Zeiss, Germany).

### Biodistribution of various NPs in tumor-bearing mice

Tumors were established by inoculating 4T1 cells subcutaneously in Balb/C mice. When the tumors reached to 100 mm^3^, 100 μL 1% various PS were i. v. administrated. After 8 h, the biodistribution of Nile Red in mice were imaged using a living imaging system (IVIS Spectrum, PerkinElmer). In order to avoid the influence of strong spontaneous fluorescence of the surrounding hairs, the tumor region was delineated as ROI separately, and this ROI region was employed to analyze the fluorescence distribution in tumor using the Living image software 4.3. After imaging, the mice were sacrificed to harvest the main organs and tumor for ex vivo imaging using this living imaging system.

### Biodistribution of Various NPs in mice with zymosan induced acute peritonitis

Male Balb/C mice were i.p. injected with 1 mL of zymosan suspension in saline (1 mg/mL) to induce acute peritonitis, then randomly allocated into 4 groups. After 16 h, mice in various groups were respectively i. v. injected with 100 μL 1% various PSs or saline as control. After 8 h of the injection, the biodistribution of Nile Red in mice were imaged using a living imaging system (IVIS Spectrum, PerkinElmer). In order to avoid the influence of strong spontaneous fluorescence of the surrounding hairs, the abdominal region was delineated as ROI separately, and this ROI region was employed to analyze the fluorescence distribution in tumor using the Living image software 4.3.

### Treatment of peritonitis

For zymosan induced acute peritonitis, male Balb/C mice were i.p. injected with 1 mL of zymosan suspension in saline (1 mg/mL) to induce acute peritonitis, then randomly allocated into 4 groups. After 12 h, mice in various groups were respectively i. v. injected with 100 μL 1% various PS or saline as control. After the injection, the survival status and survival rate of mice were recorded. To quantify the inflammatory cytokines in abdominal dropsy, 100 μL abdominal dropsy was exacted and collected after mice treated with NPs for 4 h (injected with zymosan for 4 h) via a sterile syringe. After centrifugation of dropsy at 10,621*g* for 15 min at 4 °C, the concentration of TNF-α, IL-1β, IL-10 and TGF-β in the supernatant were measured by ELISA under the guide of kit instructions. Similar treatment was conducted for male Balb/C mice with acetic acid induced acute peritonitis, which was induced by i.p. injection of 0.1 mL acetic acid solution in saline (3%).

### Treatment of tumor

Tumors were established by inoculating 4T1 cells subcutaneously in Balb/C mice. After 2 weeks, when the tumors reached to 100 mm^3^, 100 μL 1% various PS were i. v. administrated every 4 days. After 16 days, the mice were sacrificed, and the tumor tissues were isolated and divided into two parts. To quantify the inflammatory cytokines in tumor, 100 μg tumor tissue was homogenate for evaluating of the levels of inflammatory cytokines. In addition, immunofluorescence analysis was also conducted to examine expression of iNOS and CD163 in the tumor, using a Zeiss LSM confocal system.

### Statistical analysis

All data are expressed as mean ± standard deviation (SD) and analyzed by the software of SPSS 18.0 statistical package. For experiment with two groups, an unpaired t-test was performed in statistical analyses of independent continuous variables, while one-way ANOVA test with two-tailed Student’s t-test was employed in experiments with three or more than three groups. Statistical significance was assessed at p < 0.05.

## Supplementary information

**Additional file 1: Fig. S1.** Characterization of nanoparticles. (a–c) Representative TEM images of PS (a), COOH-PS (b) and DEX-PS (c). (d) Infrared spectra analysis of various PS. (e) Representative fluorescent images of various PS. (f) Size distribution of PS determined by DLS. (g) Zeta potential of PS. Scale bars in (a–c) mean 500 nm. Data in (g) are presented as the mean ± SD. n = 3, * means p < 0.05. **Fig. S2.** (a) Cell toxicity of PS, COOH-PS and DEX-PS to Raw 264.7 cells for 24 h. (b) Fluorescence intensity of PS, COOH-PS and DEX-PS at 0.01 mg/mL determined by a fluorescence spectrophotometer. (c) The uptake rates of various nanoparticles by Raw 264.7 cells at various time points. Data are presented as the mean ± SD. n = 3. **Fig. S3.** (a, b) Cell surface expression of the M1 marker (iNOS and CD86) and the M2 marker (MMR and CD163) as analyzed by flow cytometry. (c) Representative phase contrast images of Raw 264.7 cells and polarized macrophages, bar indicated 20 μm (d) Release of TNF-α, IL-1β, IL-10 and TGF-β in cell culture media was analyzed by using ELISA. (e–f) The mRNA level of pro-inflammatory cytokines (e) and anti-inflammatory cytokines (f) expressed by various cells determined by PCR. Data are presented as the mean ± SD. n = 3. **Fig. S4.** The mean fluorescence of whole cells in BMDM-M1 cells (a) and in BMDM-M2 cells (b) after incubation with various NPs. Data are presented as the mean ± SD. n = 3, * means p < 0.05. **Fig. S5.** The expression changes of MMR on M1 macrophages after incubating with nanoparticles for 4 h. Data are presented as the mean ± SD. n = 3, * means p < 0.05. **Fig. S6.** (a, b) The uptake rate of PS (a) and COOH-PS (b) by M1 macrophages influenced by various inhibitors. (c, d) The uptake rate of PS (c) and COOHPS (d) by M2 macrophages influenced by various inhibitors. Data are presented as the mean ± SD. n = 3, * means p < 0.05. **Fig. S7.** (a) The percent of Nile Red^+^ cells in MMR^+^ and MMR^−^ cells. (b) The fluorescence intensity of MMR^+^ and MMR^−^ cells. Data are presented as the mean ± SD. n = 3, * means p < 0.05. **Fig. S8.** (a) Quantification illustrating distribution of Nile Red fluorescence signals in isolated whole blood. (b) Fluorescence intensity of eight hours after injection of PSs nanoparticles in vivo. Data are presented as the mean ± SD. n = 3, * means p < 0.05. **Fig. S9.** (a) Representative confocal laser-scanning microscopy images of the M1 marker (iNOS) and M2 marker (CD163) expression in 4T1 tumor. (b) The ratio of CD86 (M1 marker)/CD206 (M2 marker) in acute peritonitis induced by zymosan. Data are presented as the mean ± SD. n = 3, * means p < 0.05. **Fig. S10.** (a) Quantitative statistical results of NPs distribution in tumor. Data are presented as the mean ± SD (n = 2 in saline group, while n = 3 in other groups). (b) Quantitative statistical results of NPs distribution in acute peritonitis lesion. Data are presented as the mean ± SD (n = 3). (c) The ex vivo fluorescent images of heart, liver, lung, kidney and spleen. (d) Quantitative statistical results of NPs distribution in major organs. Data are presented as the mean ± SD (n = 3), * means p < 0.05. **Fig. S11.** The percent of CD11b^+^ B220^−^ cells in Nile Red^+^ blood cells. Data are presented as the mean ± SD. n = 3, * means p < 0.05. **Fig. S12.** The uptake rate of PS (a) and COOH-PS (b) by Raw 264.7 cells influenced by various inhibitors. Data are presented as the mean ± SD. n = 3, * means p < 0.05. **Fig. S13.** The expression changes of CD86 on M1 macrophages after treated with various nanoparticles. Data are presented as the mean ± SD. n = 3. **Fig. S14.** The survival time of mice in zymosan induced acute peritonitis. Data are presented as the mean ± SD. n = 10, * means p < 0.05. **Fig. S15.** Growth of subcutaneous 4T1 tumors after treated with various PS. Data are presented as the mean ± SD. n = 5. **Fig. S16.** The distribution of nanoparticles in tumor observed by CLSM.

## Data Availability

The datasets used and analysed during the current study are available from the corresponding author on reasonable request.
