# Supplementary material for: The double-edged sword effect of macrophage targeting delivery system in different macrophage subsets related diseases
Source: J Nanobiotechnology. 2020 Nov 16;18:168. doi: 10.1186/s12951-020-00721-3 (PMC7667812; doi:10.1186/s12951-020-00721-3)
Supplement: Supplementary file 1 — Additional file 1: Fig. S1. Characterization of nanoparticles. (a–c) Representative TEM images of PS (a), COOH-PS (b) and DEX-PS (c). (d) Infrared spectra analysis of various PS. (e) Representative fluorescent images of various PS. (f) Size distribution of PS determined by DLS. (g) Zeta potential of PS. Scale bars in (a–c) mean 500 nm. Data in (g) are presented as the mean ± SD. n = 3, * means p < 0.05. Fig. S2. (a) Cell toxicity of PS, COOH-PS and DEX-PS to Raw 264.7 cells for 24 h. (b) Fluorescence intensity of PS, COOH-PS and DEX-PS at 0.01 mg/mL determined by a fluorescence spectrophotometer. (c) The uptake rates of various nanoparticles by Raw 264.7 cells at various time points. Data are presented as the mean ± SD. n = 3. Fig. S3. (a, b) Cell surface expression of the M1 marker (iNOS and CD86) and the M2 marker (MMR and CD163) as analyzed by flow cytometry. (c) Representative phase contrast images of Raw 264.7 cells and polarized macrophages, bar indicated 20 μm (d) Release of TNF-α, IL-1β, IL-10 and TGF-β in cell culture media was analyzed by using ELISA. (e–f) The mRNA level of pro-inflammatory cytokines (e) and anti-inflammatory cytokines (f) expressed by various cells determined by PCR. Data are presented as the mean ± SD. n = 3. Fig. S4. The mean fluorescence of whole cells in BMDM-M1 cells (a) and in BMDM-M2 cells (b) after incubation with various NPs. Data are presented as the mean ± SD. n = 3, * means p < 0.05. Fig. S5. The expression changes of MMR on M1 macrophages after incubating with nanoparticles for 4 h. Data are presented as the mean ± SD. n = 3, * means p < 0.05. Fig. S6. (a, b) The uptake rate of PS (a) and COOH-PS (b) by M1 macrophages influenced by various inhibitors. (c, d) The uptake rate of PS (c) and COOHPS (d) by M2 macrophages influenced by various inhibitors. Data are presented as the mean ± SD. n = 3, * means p < 0.05. Fig. S7. (a) The percent of Nile Red+ cells in MMR+ and MMR− cells. (b) The fluorescence intensity of MMR+ and MMR− [file 12951_2020_721_MOESM1_ESM.docx]

**Additional file**

**The Double-Edged Sword effect of Macrophage Targeting Delivery System in Different Macrophage Subsets Related Diseases**

*Yuchuan Yuan, Ling Long, Jiaxing Liu, Yongyao Lin, Cuiping Peng, Yue Tang, Xuemei Zhou, Shuhui Li, Chengyuan Zhang *, Xiaohui Li *, Xing Zhou **

1. **Supplementary experimental section**

***1.1 The Characterization of nanoparticles***

The hydrodynamic diameter and distribution of various nanoparticles were measured by a Malvern Zetasizer Nano ZS instrument at 25°C. The morphology of them was observed by transmission electron microscopy (TEM) on a Tecnai-10 microscope (Philips, the Netherlands) operating at an acceleration voltage of 80 kV. The fluorescent property of nanoparticles was characterized by confocal laser scanning microscopy (CLSM) observation using a Zeiss LSM510 laser scanning confocal microscope and HITACHI fluorescence spectrophotometer (F-7000).

***1.2 Examination of the dextran consumption in dextran -PS systhesis reaction system.***

product dextran -PS was synthesized and centrifuged, the supernatant was collected carefully after centrifuge. Then the obtained supernatant was mixed with 80% phenol and H_2_SO_4_ in a 1: 2: 8 ratio. After mixing solution on a vortex test tube mixer, the absorbance at 490nm of sample was read using a spectrophotometer. The amount of residual dextran-NH_2_ in supernatant could be conducted after substitute absorbance value into the standard curve equation. According to the mount of residual dextran-NH_2_ in supernatant, the amout of dextran linked to COOH-PS was cropsssondgly caculated .

***1.3 The mechanisms of internalization of*** ***various nanoparticles by macrophages***

Various macrophages were seeded in 2 mL growth medium with a density of 5×10^5^ cells/well in 6-well plates for 24 hours. Before 4 hours various polystyrene nanoparticles added, these cells were pre-incubated with various specific pharmacological pathway inhibitors of macropinocytosis (1 μg/mL rottlerin for 30 min), pinocytosis (2 μM colchicine for 2 h), phagocytosis (10 μg/mL cytochalasin B for 2 h), clathrin-mediated endocytosis (67 μg/mL monodansyl cadaverine for 10 min), caveolae-mediated endocytosis (50 μg/mL nystatin for 15 min), dynamin-dependent endocytosis (10 μM dynasore for 30 min) [1], scavenger receptors dependent uptake (10 μg/mL Polyinosinic acid for 30 min), [2] mannan receptor dependent uptake (200 μg/mL mannan for 20 min),[3] Dectin-1 dependent uptake (100 μg/mL laminarin for 20 min).[4]

***1.4 Cytoxicity of nanoparticles on Raw 264.7 cells***

Murine monocyte/macrophage Raw 264.7 cells were cultured in 100 μL growth medium at a density of 1.0 × 10^5^ cells/well in 96-well plates. After 24 hours, PS, COOH-PS, DEX-PS nanoparticles at pre-determined concentration was added in cell suspension for 12 hour incubation. After treatment, the cell viability of treated cells was quantified using a CCK-8 kit (Med Chem Express, Monmouth Junction, USA).

***1.5 Induction of Polarized macrophages from Raw 264.7 cells***

For induction of M1 macrophages, Raw 264.7 cells were incubated with LPS (1 μg/mL) plus IFN-γ (15 ng/mL) for 12 hours. Then, these cells were washed with serum-free RPMI 1640 medium for three times, and cultured in this medium for 48 h. Followed a 48 h serum starvation, these polarized cells were collected as M1 macrophages. For induction of M2 macrophage, Raw 264.7 cells were cultured in complete medium with 10 % FBS and 15 ng/mL IL-4. After 48 hours, these cells were washed with serum-free DMEM for three times, and then cultured in this medium for 48 h. Followed a 48 h serum starvation, these polarized cells were collected as M2 macrophages. The markers of different macrophage phenotypes expression in cells, such as CD206, CD163, CD86, iNOS, was measured by flow cytometry. In addition, the cytokines produced from Raw 264.7, M1 macrophage and M2 macrophage in the medium, such as TNF-α, IL-1β, TGF-β and IL-10, was determined by qPCR assay and enzyme-linked immunosorbent assay (ELISA) kits (R&D, Minneapolis, MN). The cell morphology of M1 macrophage and M2 macrophage was observed under inverted microscope.

***1.6 Extraction and polarization of bone-marrow derivated macrophages.***

Mice were sacrificed by cervical dislocation. The whole tibia was excised, and both tibial extremities were cut to remove the bone marrow from the diaphysis by flushing with cold PBS. Bone-marrow cells were washed with PBS twice and cultured with complete 1640 medium under the presence of 20 ng/mL G-CSF to harvest bone-marrow derivated macrophsges (BMDM). BMDM-M1 macrophages were harvested by culturing BMDM with 100 ng/ mL LPS and 20ng/ mL IFN-gamma in complete 1640 medium for 12 hours; BMDM-M2 macrophages were harvested by culturing BMDM with 20 ng/ mL IL-4 and 20ng/ mL IL-13 in complete 1640 medium for 24 hours.

1. **Supplementary Figures**


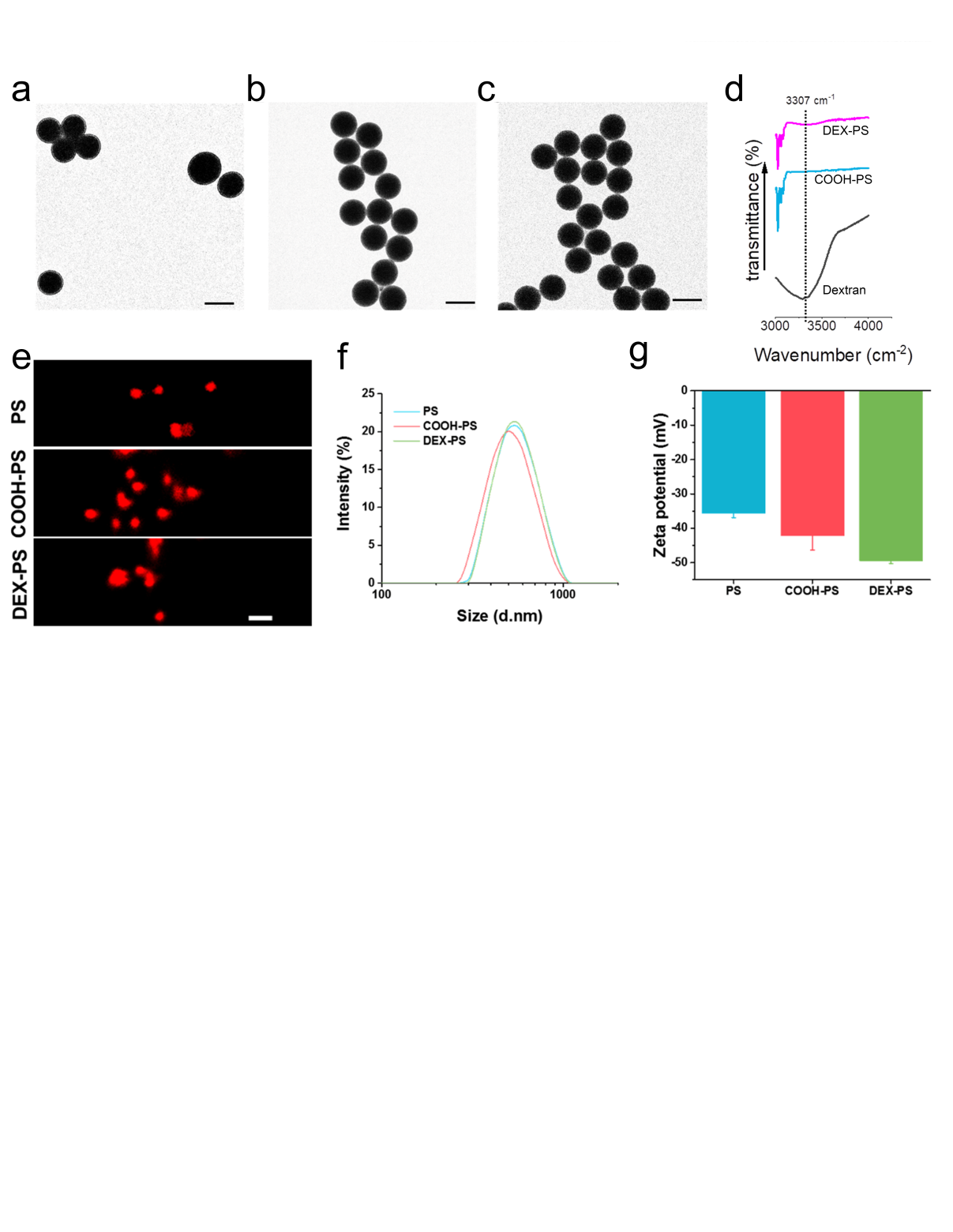


**Fig. S1** Characterization of nanoparticles. (a-c) Representative TEM images of PS (a), COOH-PS (b) and DEX-PS (c). (d) Infrared spectra analysis of various PS. (e) Representative fluorescent images of various PS. (f) Size distribution of PS determined by DLS. (g) Zeta potential of PS. Scale bars in (a-c) mean 500 nm. Data in (g) are presented as the mean ± SD. n=3. * means p<0.05.

**Note:** As shown in TEM images, dextran functionalized polystyrene nanoparticles (DEX-PS), carboxyl-functionalized polystyrene nanoparticles (COOH-PS) and un-functionalized polystyrene nanoparticles (PS) all presented a uniform and regular nano-spherical structure (Figure S1a). Notably, PS, COOH-PS and DEX-PS showed similar fluorescence properties and could be clearly observed under confocal microscopy (Figure S1b). Additionally, the size of PS, COOH-PS and DEX-PS were similar (Figure S1c). Zeta potentials of different nanoparticles were all negative (Figure S1g).


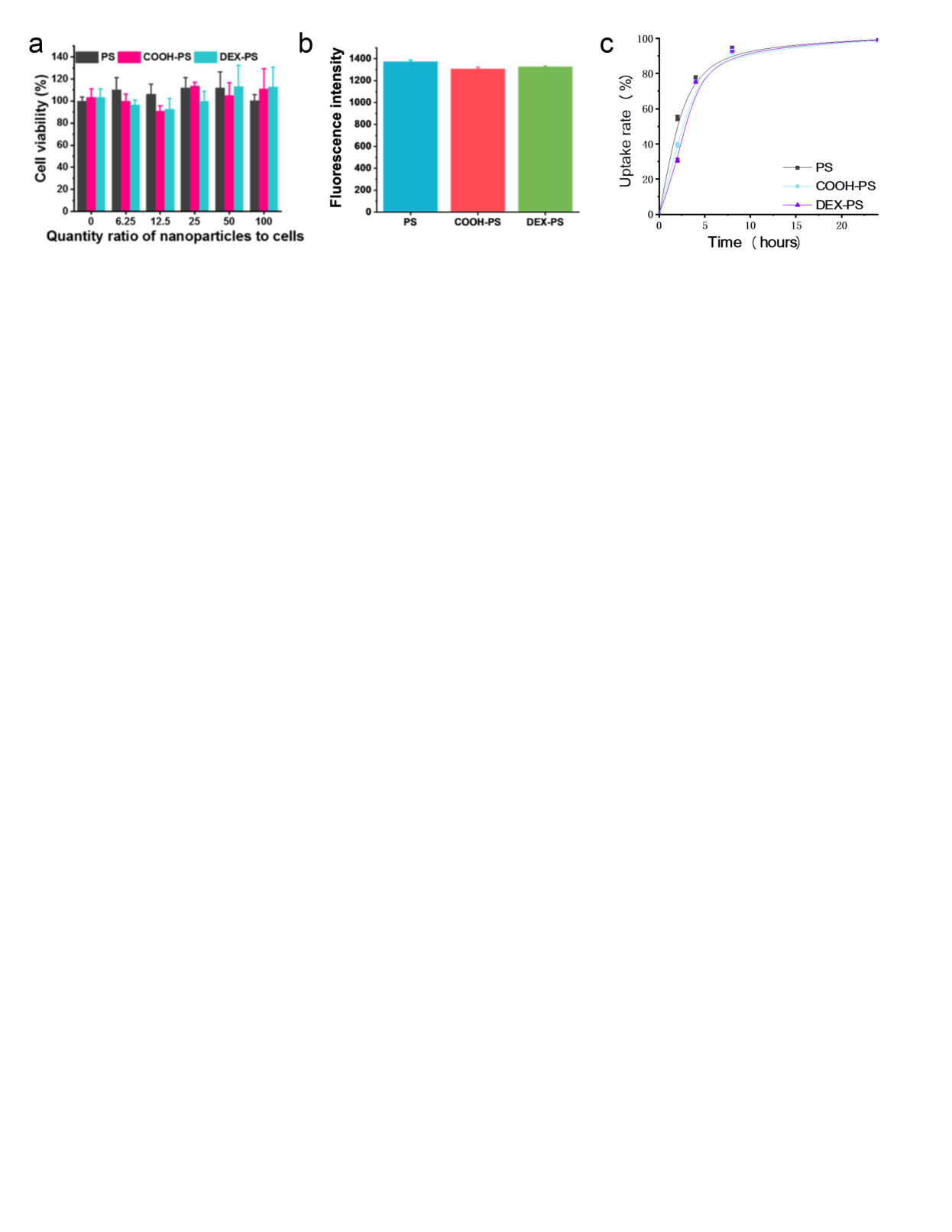


**Fig. S2** (a) Cell toxicity of PS, COOH-PS and DEX-PS to Raw 264.7 cells for 24 hours. (b) Fluorescence intensity of PS, COOH-PS and DEX-PS at 0.01 mg/mL determined by a fluorescence spectrophotometer. (c) The uptake rates of various nanoparticles by Raw 264.7 cells at various time points. Data are presented as the mean ± SD. n=3.

**
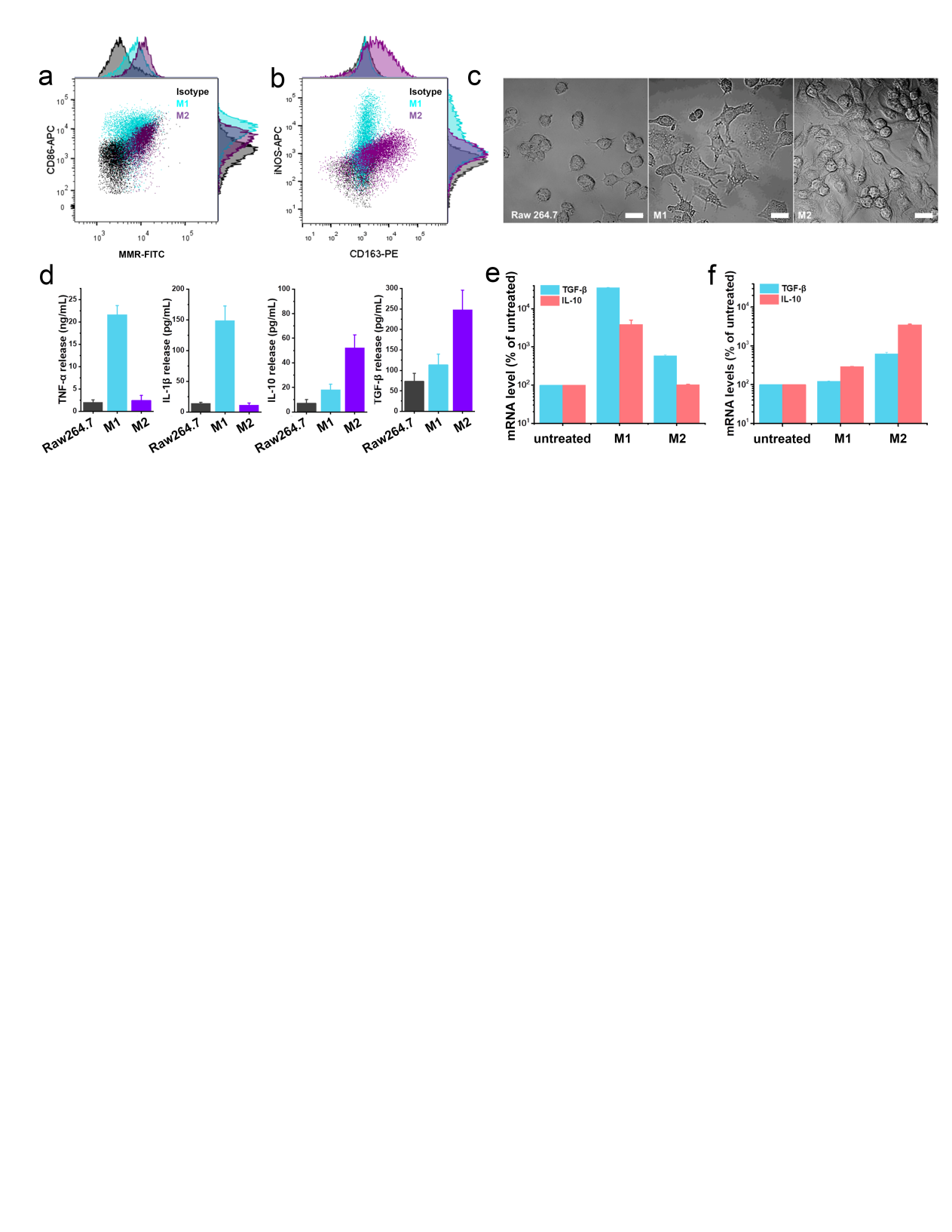
**

**Fig. S3** (a, b) Cell surface expression of the M1 marker (iNOS and CD86) and the M2 marker (MMR and CD163) as analyzed by flow cytometry. (c) Representative phase contrast images of Raw 264.7 cells and polarized macrophages, bar indicated 20 μm (d) Release of TNF-α, IL-1β, IL-10 and TGF-β in cell culture media was analyzed by using ELISA. (e-f) The mRNA level of pro-inflammatory cytokines (e) and anti-inflammatory cytokines (f) expressed by various cells determined by PCR. Data are presented as the mean ± SD. n=3


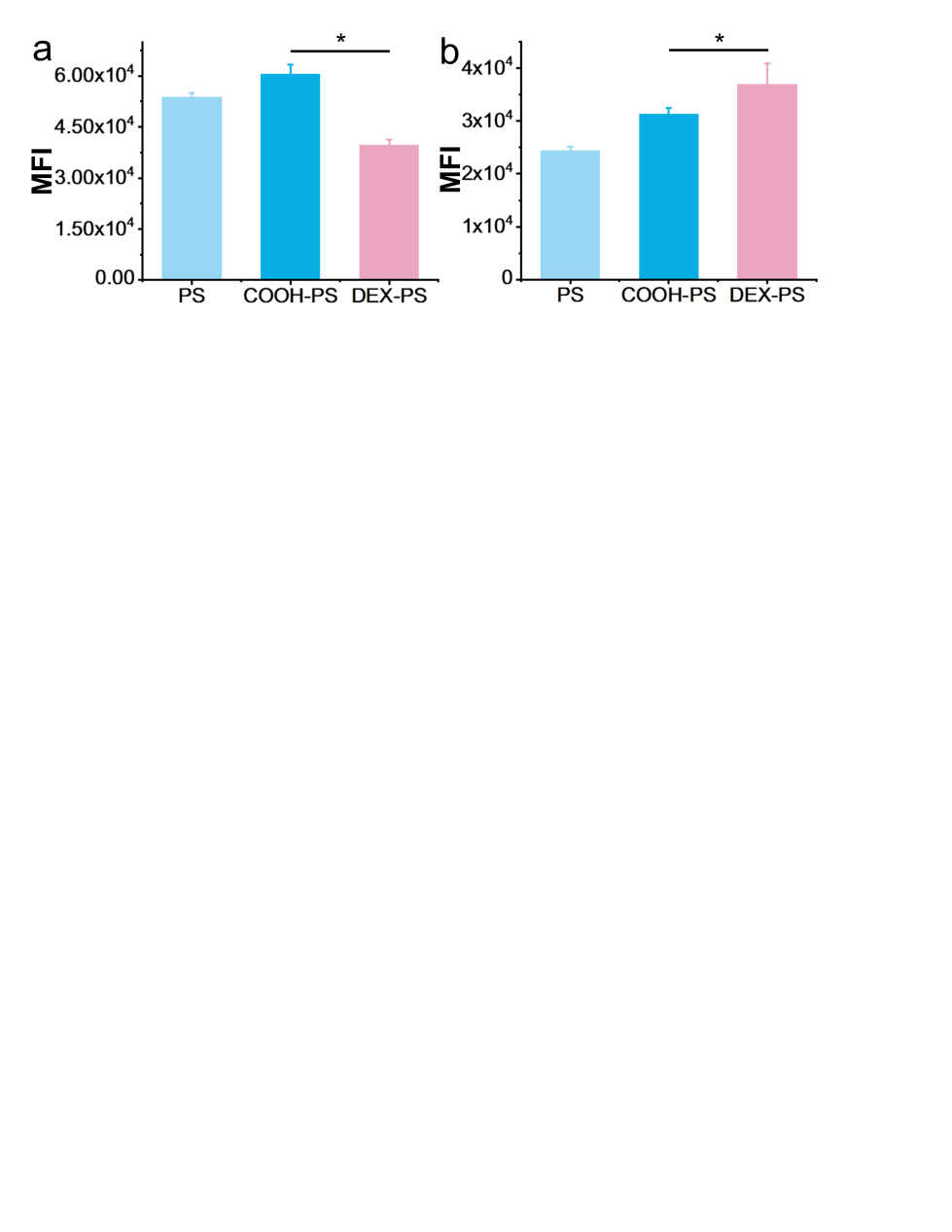


**Fig. S4** The Mean fluorescence of whole cells in BMDM-M1 cells (a) and in BMDM-M2 cells (b) after incubation with various NPs. Data are presented as the mean ± SD. n=3. * means p<0.05.


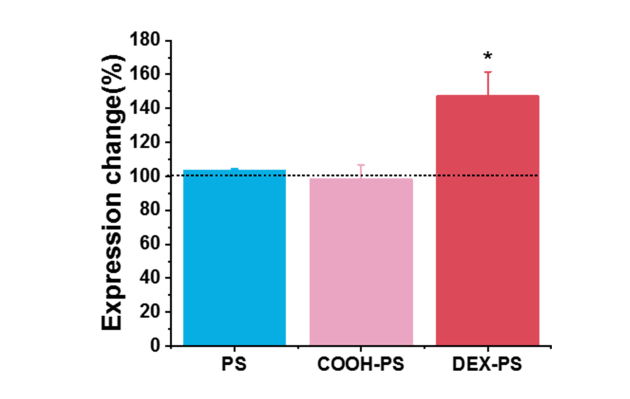


**Fig. S5** The expression changes of MMR on M1 macrophages after incubating with nanoparticles for 4 hours. Data are presented as the mean ± SD. n=3. * means p<0.05.


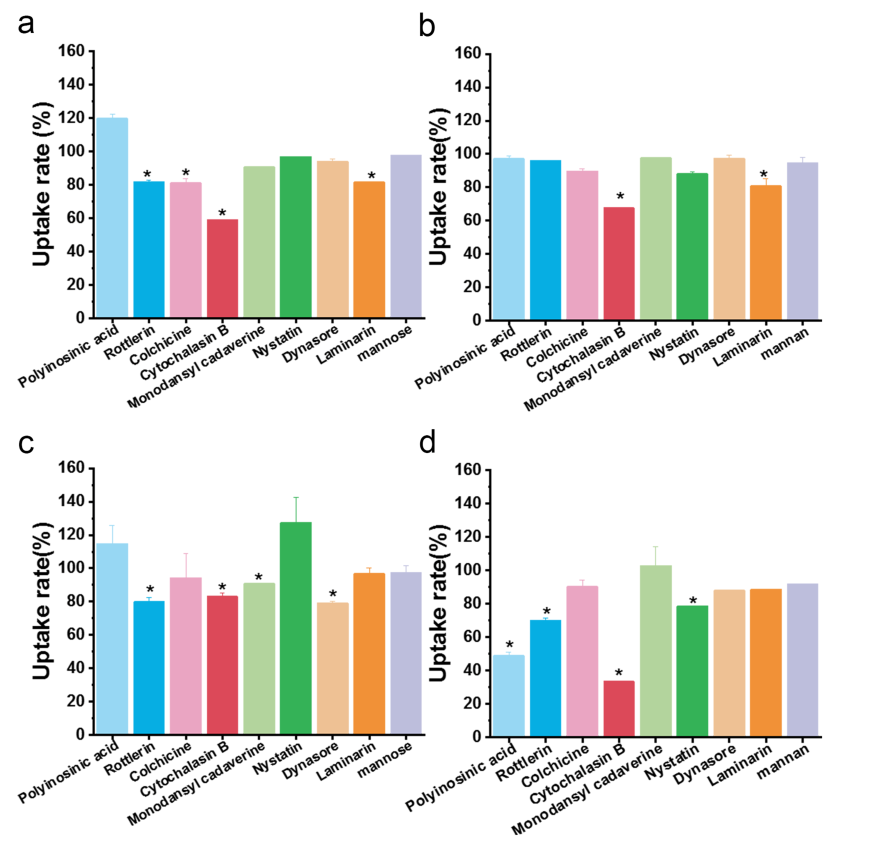


**Fig. S6** (a, b) The uptake rate of PS (a) and COOH-PS (b) by M1 macrophages influenced by various inhibitors. (c, d) The uptake rate of PS (c) and COOH-PS (d) by M2 macrophages influenced by various inhibitors. Data are presented as the mean ± SD. n=3. * means p<0.05.


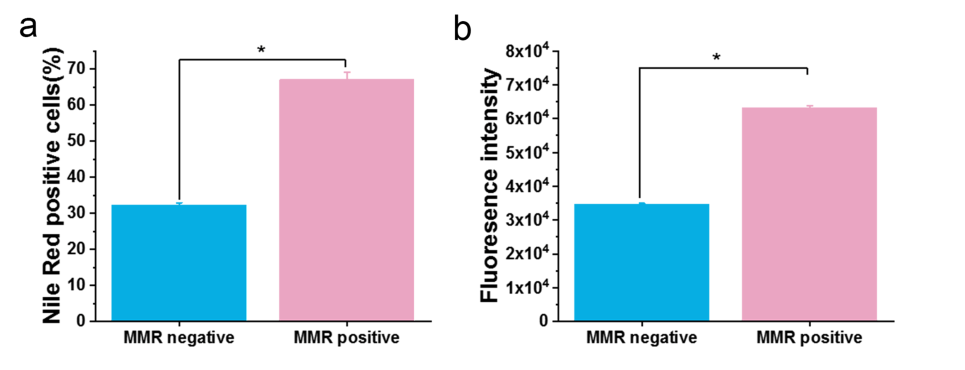


**Fig. S7** (a) The percent of Nile Red^+^ cells in MMR^+^ and MMR^-^ cells. (b) The fluorescence intensity of MMR^+^ and MMR^-^ cells. Data are presented as the mean ± SD. n=3. * means p<0.05.


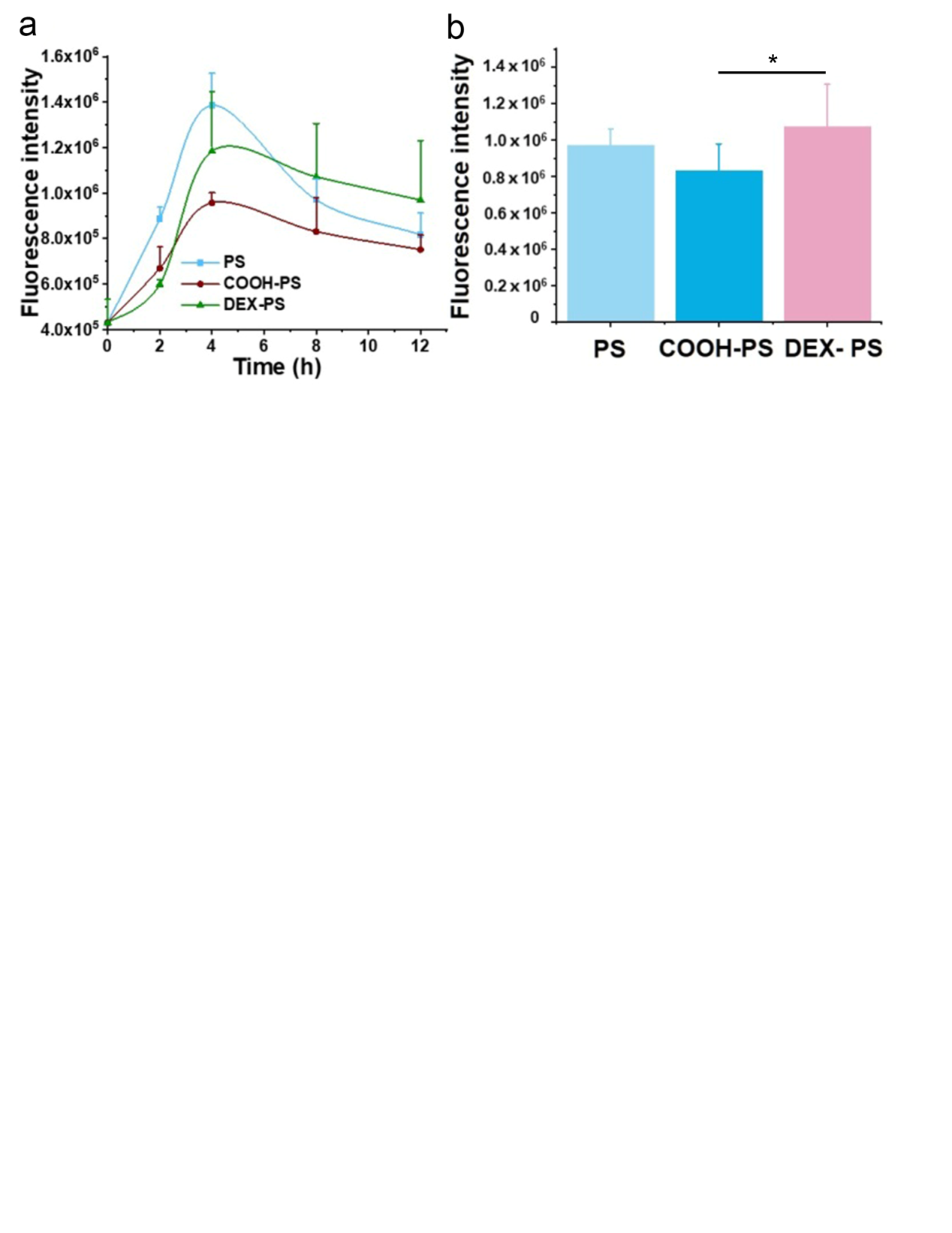


**Fig. S8** (a) Quantification illustrating distribution of Nile Red fluorescence signals in isolated whole blood. (b) Fluorescence intensity of eight hours after injection of PSs nanoparticles *in vivo.* Data are presented as the mean ± SD. n=3. * means p<0.05.


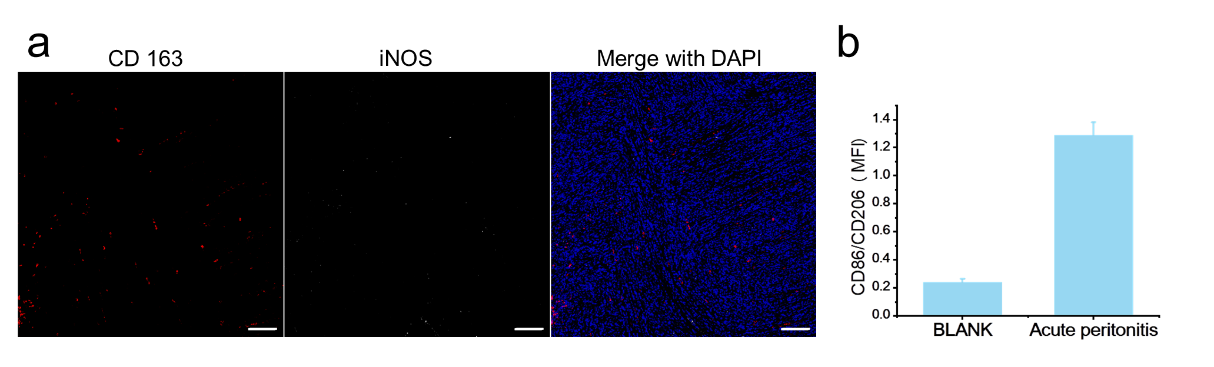

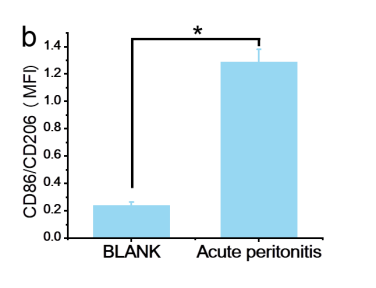


Fig. S9 (a) Representative confocal laser-scanning microscopy images of the M1 marker (iNOS) and M2 marker (CD163) expression in 4T1 tumor. (b)The ratio of CD86 (M1 marker) /CD206 (M2 marker) in acute peritonitis induced by zymosan. Data are presented as the mean ± SD. n=3. * means p<0.05.


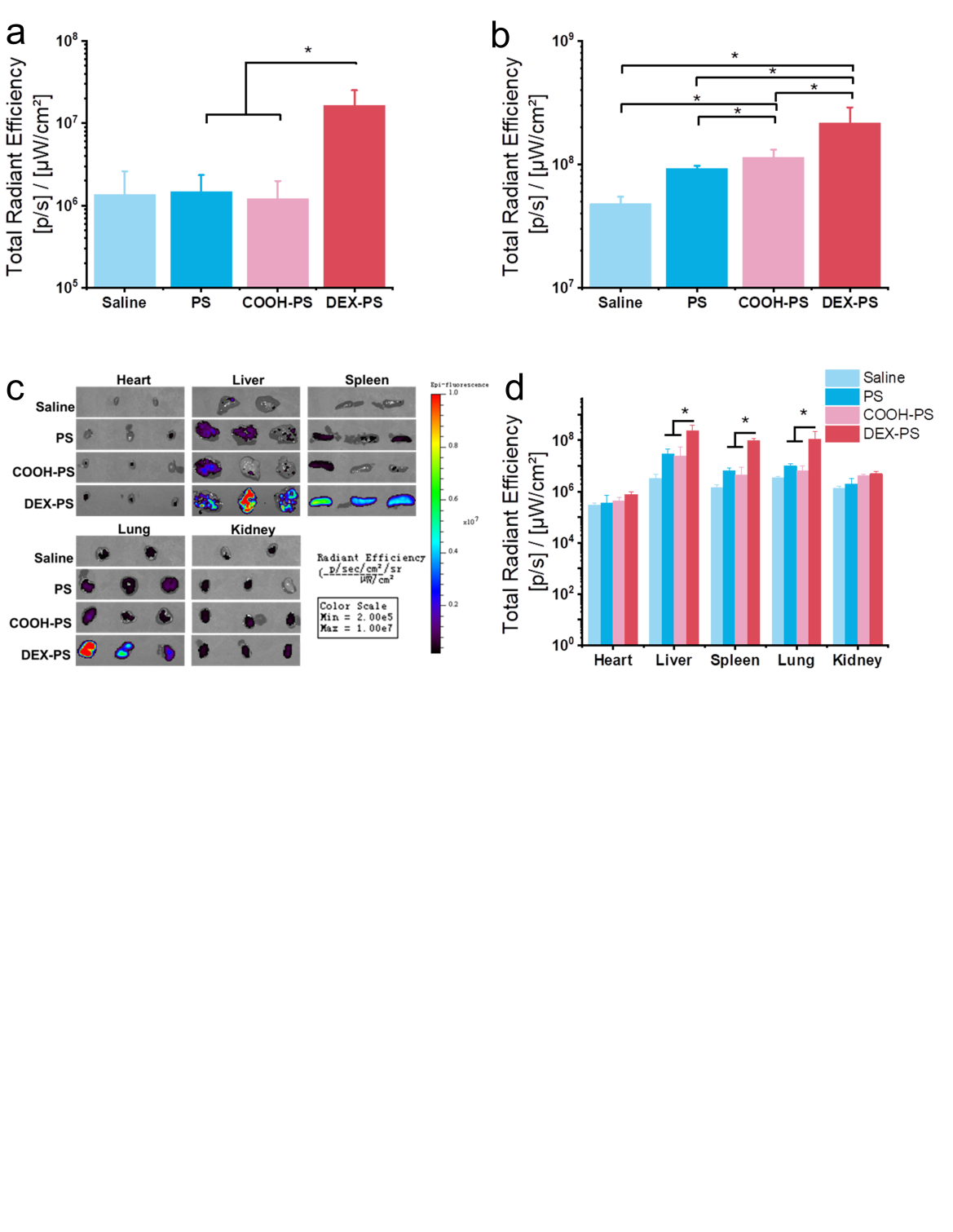


**Fig. S10** (a) Quantitative Statistical results of NPs distribution in tumor. Data are presented as the mean ± SD (n=2 in saline group, while n=3 in other groups). (b) Quantitative Statistical results of NPs distribution in acute peritonitis lesion. Data are presented as the mean ± SD (n=3). (c) The *ex vivo* fluorescent images of heart, liver, lung, kidney and spleen.(d) Quantitative Statistical results of NPs distribution in major organs. Data are presented as the mean ± SD (n=3). * means p<0.05


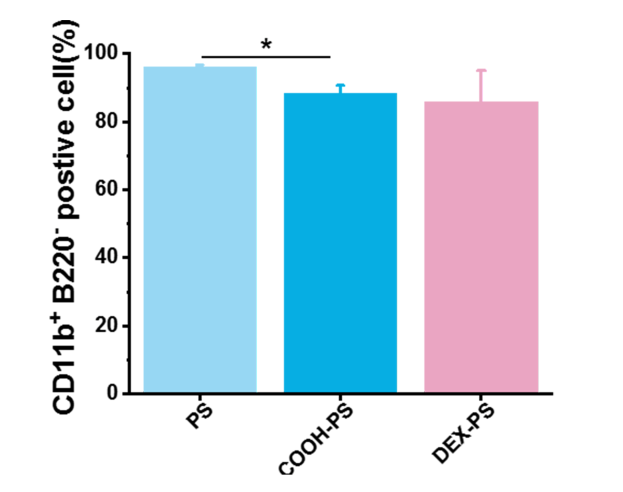


**Fig. S11** The percent of CD11b^+^ B220^-^ cells in Nile Red^+^ blood cells. Data are presented as the mean ± SD. n=3. * means p<0.05.


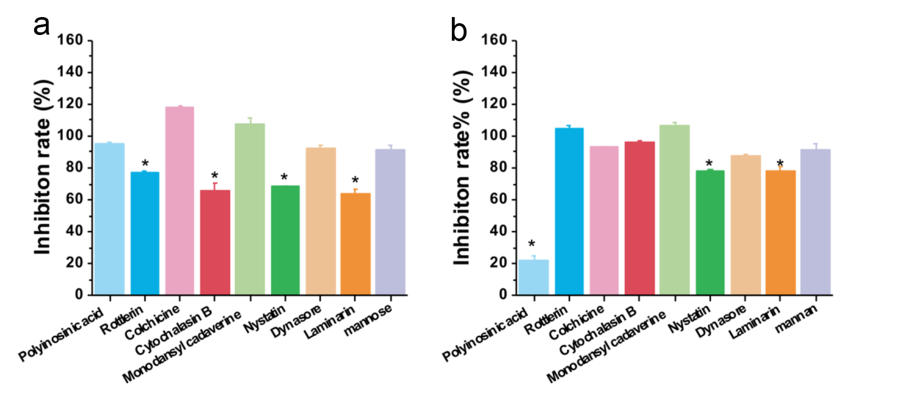


**Fig. S12** The uptake rate of PS (a) and COOH-PS (b) by Raw 264.7 cells influenced by various inhibitors. Data are presented as the mean ± SD. n=3. * means p<0.05.


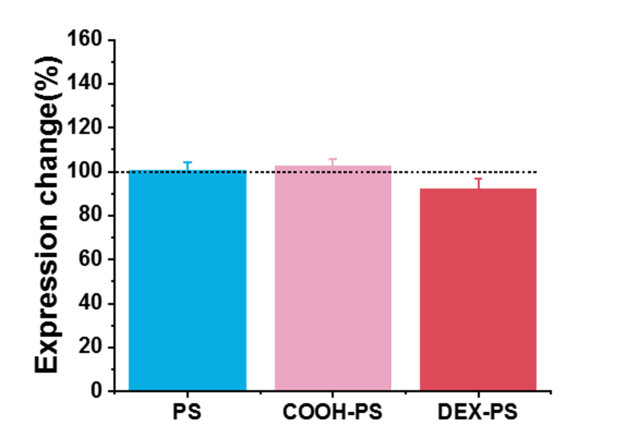


**Fig. S13** The expression changes of CD86 on M1 macrophages after treated with various nanoparticles. Data are presented as the mean ± SD. n=3


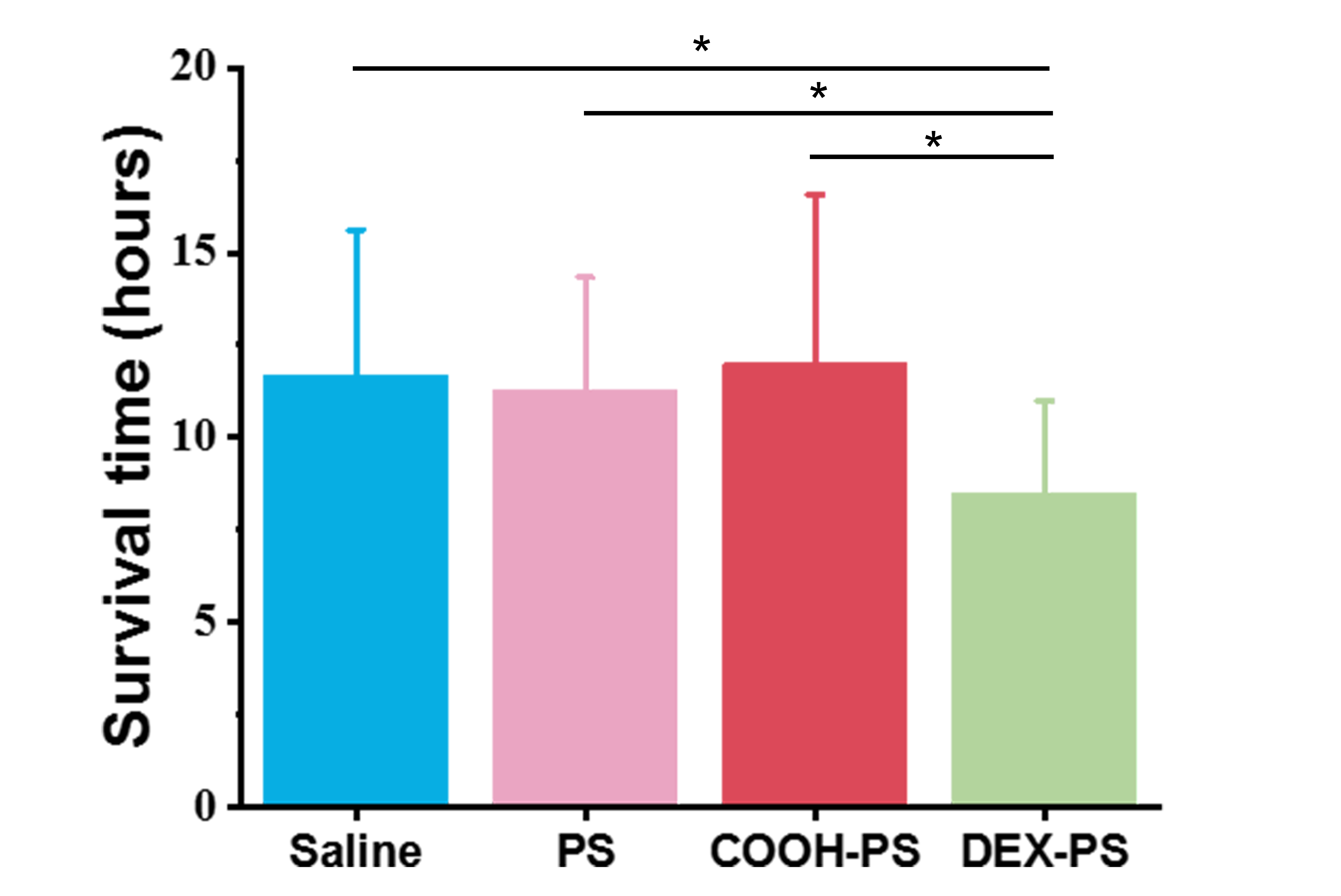


**Fig. S14** The survival time of mice in zymosan induced acute peritonitis. Data are presented as the mean ± SD. n=10. * means p<0.05.


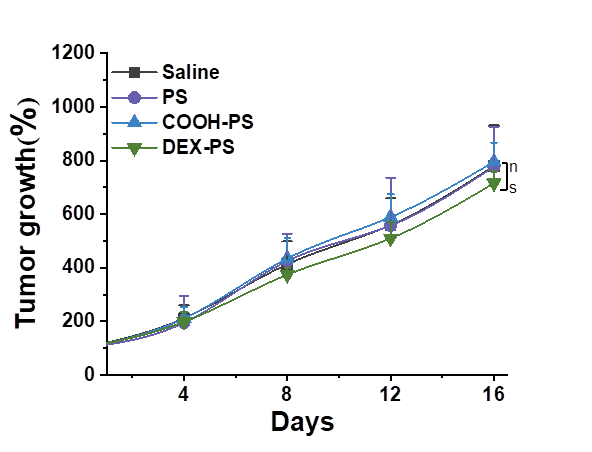


**Fig. S15** Growth of subcutaneous 4T1 tumors after treated with various PS. Data are presented as the mean ± SD. n=5.


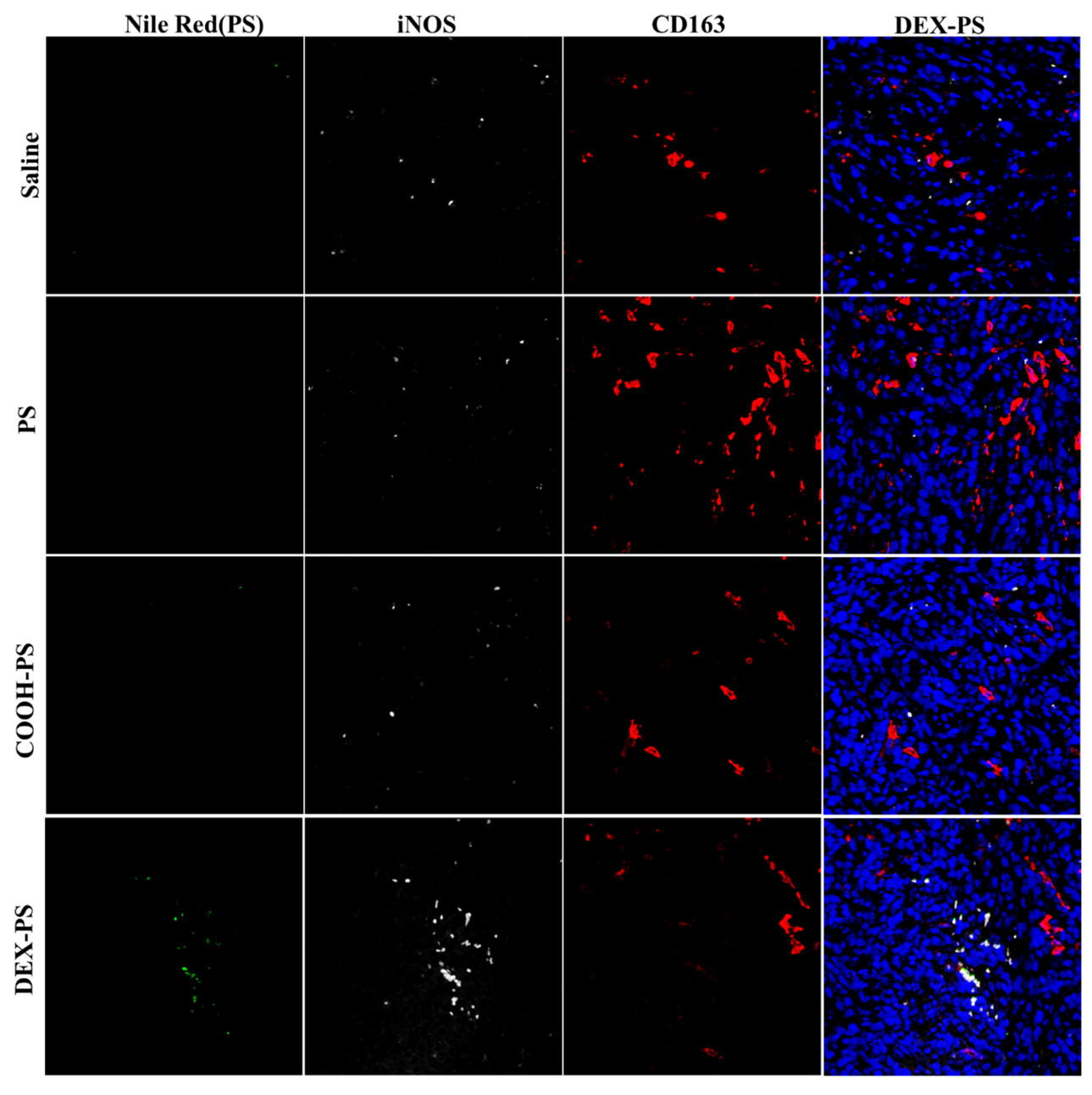


**Fig. S16** The distribution of nanoparticles in tumor observed by CLSM.

REFERENCES

[1] Lunov O, Syrovets T, Loos C, Beil J, Delacher M, Tron K, Nienhaus GU, Musyanovych A, Mailander V, Landfester K, Simmet T. Differential uptake of functionalized polystyrene nanoparticles by human macrophages and a monocytic cell line. ACS Nano 2011; 3: 1657-1669.

[2] Rice PJ, Kelley JL, Kogan G, Ensley HE, Kalbfleisch JH, Browder IW, Williams DL. Human monocyte scavenger receptors are pattern recognition receptors for (1→ 3)‐β‐D‐glucans. J. Leukocyte Biol. 2002; 1: 140-146.

[3] Chao Y, Karmali PP, Simberg D. Advances in Experimental Medicine and Biology. In: Zahavy E, Ordentlich A, Yitzhaki S, Shafferman A. (eds) Dordrecht: Springer; 2012.

[4] Brown GD, Taylor PR, Reid DM, Willment JA, Williams DL, Martinez-Pomares L, Wong SYC, Gordon S. Dectin-1 Is A Major β-Glucan Receptor On Macrophages. J. Exp. Med. 2002; 3: 407-412.
